# Computing Generalized Convolutions Faster Than Brute Force

**DOI:** 10.1007/s00453-023-01176-2

**Published:** 2023-10-06

**Authors:** Barış Can Esmer, Ariel Kulik, Dániel Marx, Philipp Schepper, Karol Węgrzycki

**Affiliations:** 1 CISPA Helmholtz Center for Information Security, Saarbrücken, Germany; 2 Max Planck Institute for Informatics, Saarland University, Saarbrücken, Germany

**Keywords:** Generalized Convolution, Fast Fourier Transform, Fast Subset Convolution, Orthogonal Vectors, Theory of computation, Parameterized complexity and exact algorithms, Theory of computation, Algorithm design techniques

## Abstract

In this paper, we consider a general notion of convolution. Let $D$ be a finite domain and let $D^n$ be the set of *n*-length vectors (tuples) of $D$. Let $f :D\times D\rightarrow D$ be a function and let $\oplus _f$ be a coordinate-wise application of *f*. The $f$-Convolution of two functions $g,h :D^n \rightarrow \{-M,\ldots ,M\}$ is

$$\begin{aligned} (g \mathbin {\circledast _{f}}h)(\textbf{v}) {:}{=}\sum _{\begin{array}{c} \textbf{v}_g,\textbf{v}_h \in D^n\\ \text {s.t. } \textbf{v}= \textbf{v}_g \oplus _f \textbf{v}_h \end{array}} g(\textbf{v}_g) \cdot h(\textbf{v}_h) \end{aligned}$$

for every $\textbf{v}\in D^n$. This problem generalizes many fundamental convolutions such as Subset Convolution, XOR Product, Covering Product or Packing Product, etc. For arbitrary function *f* and domain $D$ we can compute $f$-Convolution via brute-force enumeration in $\widetilde{{\mathcal {O}}}(|D|^{2n} \cdot \textrm{polylog}(M))$ time. Our main result is an improvement over this naive algorithm. We show that $f$-Convolution can be computed exactly in $\widetilde{{\mathcal {O}}}( (c \cdot |D|^2)^{n} \cdot \textrm{polylog}(M))$ for constant $c {:}{=}3/4$ when $D$ has even cardinality. Our main observation is that a *cyclic partition* of a function $f :D\times D\rightarrow D$ can be used to speed up the computation of $f$-Convolution, and we show that an appropriate cyclic partition exists for every *f*. Furthermore, we demonstrate that a single entry of the $f$-Convolution can be computed more efficiently. In this variant, we are given two functions $g,h :D^n \rightarrow \{-M,\ldots ,M\}$ alongside with a vector $\textbf{v}\in D^n$ and the task of the $f$-Query problem is to compute integer $(g \mathbin {\circledast _{f}}h)(\textbf{v})$. This is a generalization of the well-known Orthogonal Vectors problem. We show that $f$-Query can be computed in $\widetilde{{\mathcal {O}}}(|D|^{\frac{\omega }{2} n} \cdot \textrm{polylog}(M))$ time, where $\omega \in [2,2.372)$ is the exponent of currently fastest matrix multiplication algorithm.

## Introduction

Convolutions occur naturally in many algorithmic applications, especially in the exact and parameterized algorithms. The most prominent example is a subset convolution procedure [22, 37], for which an efficient $\widetilde{{\mathcal {O}}}(2^n \cdot \textrm{polylog}(M))$ time algorithm for subset convolution dates back to Yates [40] but in the context of exact algorithms it was first used by Björklund et al. [6].^1^ Researchers considered a plethora of other variants of convolutions, such as: Cover Product, XOR Product, Packing Product, Generalized Subset Convolution, Discriminantal Subset Convolution, Trimmed Subset Convolution or Lattice-based Convolution [6–8, 10, 11, 20, 24, 35]. These subroutines are crucial ingredients in the design of efficient algorithms for many exact and parameterized algorithms such as Hamiltonian Cycle, Feedback Vertex Set, Steiner Tree, Connected Vertex Cover, Chromatic Number, Max *k*-Cut or Bin Packing [5, 10, 19, 28, 39, 41]. These convolutions are especially useful for dynamic programming algorithms on tree decompositions and occur naturally during join operations (e.g., [19, 34, 35]). Usually, in the process of algorithm design, the researcher needs to design a different type of convolution from scratch to solve each of these problems. Often this is a highly technical and laborious task. Ideally, we would like to have a single tool that can be used as a blackbox in all of these cases. This motivates the following ambitious goal in this paper: 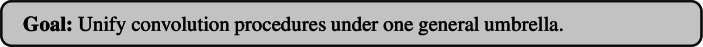 Towards this goal, we consider the problem of computing *f*-*Generalized Convolution* ($f$-Convolution) introduced by van Rooij [34]. Let $D$ be a finite domain and let $D^n$ be the *n* length vectors (tuples) of $D$. Let $f :D\times D\rightarrow D$ be an arbitrary function and let $\oplus _f$ be a coordinate-wise application of the function *f*.^2^ For two functions $g,h :D^n \rightarrow {\mathbb {Z}}$ the $f$-Convolution, denoted by $(g \mathbin {\circledast _{f}}h) :D^n \rightarrow {\mathbb {Z}}$, is defined for all $\textbf{v}\in D^n$ as

$$\begin{aligned} (g \mathbin {\circledast _{f}}h)(\textbf{v}) {:}{=}\sum _{\begin{array}{c} \textbf{v}_g,\textbf{v}_h \in D^n\\ \text {s.t. } \textbf{v}= \textbf{v}_g \oplus _f \textbf{v}_h \end{array}} g(\textbf{v}_g) \cdot h(\textbf{v}_h). \end{aligned}$$

Here we consider the standard ${\mathbb {Z}}(+,\cdot )$ ring. Through the paper we assume that *M* is the absolute value of the maximum integer given on the input.

In the $f$-Convolution problem the functions $g,h :D^n \rightarrow {\{-M,\ldots , M\}}$ are given as an input and the output is the function $(g \mathbin {\circledast _{f}}h)$. Note, that the input and output of the $f$-Convolution problem consist of $3\cdot |D|^n$ integers. Hence it is conceivable that $f$-Convolution could be solved in $\widetilde{{\mathcal {O}}}(|D|^n \cdot \textrm{polylog}(M))$. Such a result for arbitrary *f* would be a real breakthrough in how we design parameterized algorithms. So far, however, researchers have focused on characterizing functions *f* for which $f$-Convolution can be solved in $\widetilde{{\mathcal {O}}}(|D|^n \cdot \textrm{polylog}(M))$ time. In [34] van Rooij considered specific instances of this setting, where for some constant $r \in {\mathbb {N}}$ the function *f* is defined as either (i) standard addition: $f(x,y) {:}{=}x+y$, or (ii) addition with a maximum: $f(x, y) {:}{=}\min (x+y,r-1)$, or (iii) addition modulo *r*, or (iv) maximum: $f(x,y) {:}{=}\max (x,y)$. Van Rooij [34] showed that for these special cases the $f$-Convolution can be solved in $\widetilde{{\mathcal {O}}}(|D|^n \cdot \textrm{polylog}(M))$ time. His results allow the function *f* to differ between coordinates. A recent result regarding generalized Discrete Fourier Transform [32] can be used in conjunction with Yates’s algorithm [40] to compute $f$-Convolution in $\widetilde{{\mathcal {O}}}(|D|^{\omega \cdot n / 2} \cdot \textrm{polylog}(M))$ time when *f* is a finite-group operation and $\omega$ is the exponent of the currently fastest matrix-multiplication algorithms.^3^ To the best of our knowledge these are the most general settings where convolution has been considered so far.

Nevertheless, for an arbitrary function *f*, to the best of our knowledge the state-of-the-art for $f$-Convolution is a straightforward quadratic time enumeration. 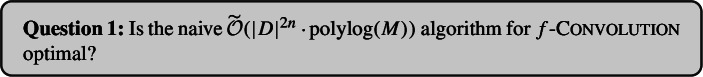 Similar questions were studied from the point of view of the Fine-Grained Complexity. In that setting the focus is on convolutions with sparse representations, where the input size is only the size of the support of the functions *g* and *h*. It is conjectured that even subquadratic algorithms are highly unlikely for these representations [18, 25]. However, these lower bounds do not answer Question 1, because they are highly dependent on the sparsity of the input.

### Our Results

We provide a positive answer to Question 1 and show an exponential improvement (in *n*) over a naive $\widetilde{{\mathcal {O}}}(|D|^{2n} \cdot \textrm{polylog}(M))$ algorithm for every function *f*.

#### Theorem 1.1

(Generalized Convolution) Let $D$ be a finite set and $f:D\times D\rightarrow D$. There is an algorithm for $f$-Convolution with the following running time $\widetilde{{\mathcal {O}}}\left( \big ( \frac{3}{4} \cdot |D|^2 \big )^{n} \cdot \textrm{polylog}(M)\right)$ when $\vert {D} \vert$ is even, or $\big (\big ( \frac{3}{4} \cdot |D|^2+\frac{1}{4}\cdot |D| \big )^{n}\big )$ when $\vert {D} \vert$ is odd.

Observe that the running time obtained by Theorem 1.1 improves upon the brute-force for every $|D| \ge 2$. Our technique works in a more general setting when $g :L^n \rightarrow {\mathbb {Z}}$ and $h:R^n \rightarrow {\mathbb {Z}}$ and $f:L \times R \rightarrow T$ for arbitrary domains *L*, *R* and *T* (see Sect. 2 for the exact running time dependence).

**Our Technique: Cyclic Partition** Now, we briefly sketch the idea behind the proof of Theorem 1.1. We say that a function is *k*-cyclic if it can be represented as an addition modulo *k* (after relabeling the entries of the domain and image). These functions are somehow simple, because as observed in [33, 34] $f$-Convolution can be computed in $\widetilde{{\mathcal {O}}}(k^n \cdot \textrm{polylog}(M))$ time if *f* is *k*-cyclic. In a nutshell, our idea is to partition the function $f:D\times D\rightarrow D$ into cyclic functions and compute the convolution on these parts independently.

**Fig. 1:**
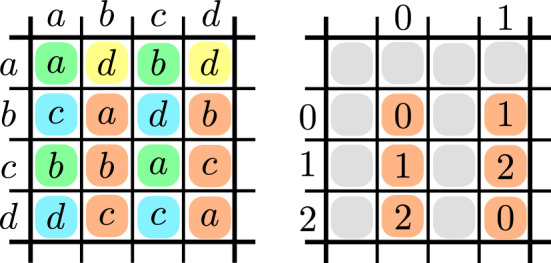
Left figure illustrates exemplar function $f :D\times D\rightarrow D$ over domain $D{:}{=}\{a,b,c,d\}$. We highlighted a cyclic partition with red, blue, yellow and blue colors. Each color represents a different minor of *f*. On the right figure we demonstrate that the red-highlighted minor can be represented as addition modulo 3 (after relabeling $a \mapsto 0$, $b\mapsto 1$ and $c \mapsto 2$). Hence the red minor has cost 3. The reader can further verify that green and blue minors have cost 2 and yellow minor has cost 1, hence the cost of that particular partition is $3+2+2+1 = 8$ (Color figure online)

More formally, a cyclic minor of the function $f:D\times D\rightarrow D$ is a (combinatorial) rectangle $A \times B$ with $A,B\subseteq D$ and a number $k\in {\mathbb {N}}$ such that *f* restricted to *A*, *B* is a *k*-cyclic function. The cost of the cyclic minor (*A*, *B*, *k*) is $\textrm{cost}(A,B) {:}{=}k$. A cyclic partition is a set $\{(A_1,B_1,k_1),\ldots ,(A_m,B_m,k_m)\}$ of cyclic minors such that for every $(a,b) \in D\times D$ there exists a unique $i \in [m]$ with $(a,b) \in A_i \times B_i$. The cost of the cyclic partition ${\mathcal {P}}= \{(A_1,B_1,k_1),\ldots ,(A_m,B_m,k_m)\}$ is $\textrm{cost}({\mathcal {P}}) {:}{=}\sum _{i=1}^m k_i$. See Fig. 1 for an example of a cyclic partition.

Our first technical contribution is an algorithm to compute $f$-Convolution when the cost of a cyclic partition is small.

#### Lemma 1.2

(Algorithm for $f$-Convolution ) Let $D$ be an arbitrary finite set, $f:D\times D\rightarrow D$ and let ${\mathcal {P}}$ be the cyclic partition of *f*. Then there exists an algorithm which given $g,h:D^n \rightarrow {\mathbb {Z}}$ computes $(g \mathbin {\circledast _{f}}h)$ in $\widetilde{{\mathcal {O}}}((\textrm{cost}({\mathcal {P}})^n + |D|^n) \cdot \textrm{polylog}(M))$ time.

The idea behind the proof of Lemma 1.2 is as follows. Based on the partition ${\mathcal {P}}$, for any pair of vectors $\textbf{u},\textbf{w}\in D^n$, we can define a type ${\varvec{p}}\in [m]^n$ such that $(\textbf{u}_i,\textbf{w}_i) \in A_{{\varvec{p}}_i} \times B_{{\varvec{p}}_i}$ for every $i \in [n]$. Our main idea is to go over each type ${\varvec{p}}$ and compute the sum in the definition of $f$-Convolution only for pairs $(\textbf{v}_g,\textbf{v}_h)$ that have type ${\varvec{p}}$. In order to do this, first we select the vectors $\textbf{v}_g$ and $\textbf{v}_h$ that are compatible with this type ${\varvec{p}}$. For instance, consider the example in Fig. 1. Whenever ${\varvec{p}}_i$ refers to, say, the red-colored minor, then we consider $\textbf{v}_g$ only if its *i*-th coordinate is in $\{b,c,d\}$ and consider $\textbf{v}_h$ only if its *i*-th coordinate is in $\{b,d\}$. After computing all these vectors $\textbf{v}_g$ and $\textbf{v}_h$, we can transform them according to the cyclic minor at each coordinate. Continuing our example, as the red-colored minor is 3-cyclic, we can represent the *i*-th coordinate of $\textbf{v}_g$ and $\textbf{v}_h$ as $\{0,1,2\}$ and then the problem reduces to addition modulo 3 at that coordinate. Therefore, using the algorithm of van Rooij [34] for cyclic convolution we can handle all pairs of type ${\varvec{p}}$ in $\widetilde{{\mathcal {O}}}((\prod _{i=1}^n k_{{\varvec{p}}_i}) \cdot \textrm{polylog}(M))$ time. As we go over all $m^n$ types ${\varvec{p}}$ the sum of $m^n$ terms is

$$\begin{aligned} \sum _{{\varvec{p}}\in [m]^n} \left( \prod _{i=1}^n k_{{\varvec{p}}_i}\right) = \left( \sum _{i=1}^m k_i\right) ^n = \textrm{cost}({\mathcal {P}})^n. \end{aligned}$$

Hence, the overall running time is $\widetilde{{\mathcal {O}}}(\textrm{cost}({\mathcal {P}})^n \cdot \textrm{polylog}(M))$. This running time evaluation ignores the generation of the vectors given as input for the cyclic convolution algorithm. The efficient computation of these vectors is nontrivial and requires further techniques that we explain in Sect. 3.

It remains to provide the low-cost cyclic partition of an arbitrary function *f*.

#### Lemma 1.3

For any finite set $D$ and any function $f:D\times D\rightarrow D$ there is a cyclic partition ${\mathcal {P}}$ of *f* such that $\textrm{cost}({\mathcal {P}}) \le \frac{3}{4} |D|^2$ when $\vert {D} \vert$ is even, or $\textrm{cost}({\mathcal {P}}) \le \frac{3}{4} |D|^2 + \frac{1}{4} |D|$ when $\vert {D} \vert$ is odd.

For the sake of presentation let us assume that $|D|$ is even. In order to show Lemma 1.3, we partition $D$ into pairs $A_1,\ldots ,A_{k}$ where $k {:}{=}|D|/2$ and consider the restrictions of *f* to $A_j \times D$ one by one. Intuitively, we partition the $D\times D$ table describing *f* into pairs of rows and give a bound on the cost of each pair. This partition allows us to encode *f* on $A_j \times D$ as a directed graph *G* with $|D|$ edges and $|D|$ vertices. We observe that directed cycles and directed paths can be represented as cyclic minors. Our goal is to partition graph *G* into such subgraphs in a way that the total cost of the resulting cyclic partition is small. Following this argument, the proof of Lemma 1.3 becomes a graph-theoretic analysis. The proof of Lemma 1.3 is included in Sect. 4. We also give an example which suggests that the constant $\frac{3}{4}$ in Lemma 1.3 cannot be improved further while using the partition of $D$ into arbitrary pairs (see Lemma 4.16).

Our method applies for more general functions $f :L \times R \rightarrow T$, where domains *L*, *R*, *T* can be different and have arbitrary cardinality. We note that a weaker variant of Lemma 1.3 in which the guarantee is $\textrm{cost}({\mathcal {P}}_f) \le \frac{7}{8} |D|^2$ is easier to attain (see Sect. 4).

**Efficient Algorithm for Convolution Query** Our next contribution is an efficient algorithm to query a single value of $f$-Convolution. In the $f$-Query problem, the input is $g,h :D^n \rightarrow {\mathbb {Z}}$ and a single vector $\textbf{v}\in D^n$. The task is to compute a value $(g \mathbin {\circledast _{f}}h)(\textbf{v})$. Observe that this task generalizes^4^ the fundamental problem of Orthogonal Vectors. We show that computing $f$-Query is much faster than computing the full output of $f$-Convolution.

#### Theorem 1.4

(Convolution Query) For any finite set $D$ and function $f:D\times D\rightarrow D$ there is a $\widetilde{{\mathcal {O}}}(|D|^{\omega \cdot n / 2} \cdot \textrm{polylog}(M))$ time algorithm for the $f$-Query problem.

Here $\widetilde{{\mathcal {O}}}(m^\omega \cdot \textrm{polylog}(M))$ is the time needed to multiply two $m \times m$ integer matrices with values in ${\{-M,\ldots , M\}}$ and currently $\omega \in [2,2.372)$ [2, 21]. Note, that under the assumption that two matrices can be multiplied in the linear in the input time (i.e., $\omega = 2$) then Theorem 1.4 runs in the nearly-optimal $\widetilde{{\mathcal {O}}}(|D|^n \cdot \textrm{polylog}(M))$ time. Theorem 1.4 is significantly faster than Theorem 1.1, which can be used to solve $f$-Query in time $\widetilde{{\mathcal {O}}}\left( \left( \frac{3}{4} \cdot |D|^2 \right) ^{n} \cdot \textrm{polylog}(M)\right)$ when $\vert {D} \vert$ is even, or $\widetilde{{\mathcal {O}}}\left( \left( \frac{3}{4} \cdot |D|^2+\frac{1}{4}\cdot |D| \right) ^{n} \cdot \textrm{polylog}(M)\right)$ when $\vert {D} \vert$ is odd. This holds true even if we plug-in the naive algorithm for matrix multiplication (i.e., $\omega = 3$). The proof of Theorem 1.4 is inspired by an interpretation of the $f$-Query problem as counting length-4 cycles in a graph.

### Related Work

Arguably, the problem of computing the Discrete Fourier Transform (DFT) is the prime example of convolution-type problems in computer science. Cooley and Tukey [17] proposed the fast algorithm to compute DFT. Later, Beth [4] and Clausen [16] initiated the study of generalized DFTs whose goal has been to obtain a fast algorithm for DFT where the underlying group is arbitrary. After a long line of works (see [31] for the survey), the currently best algorithm for generalized DFT concerning group *G* runs in ${\mathcal {O}}(|G|^{\omega /2+\epsilon })$ operations for every $\epsilon > 0$ [32].

A similar technique to ours was introduced by Björklund et al. [9]. The paper gave a characterization of lattices that admit a fast zeta transform and a fast Möbius transform.

From the lower-bounds perspective to the best of our knowledge only a naive $\Omega (|D|^n)$ lower bound is known for $f$-Convolution (as this is the output size). We note that known lower bounds for different convolution-type problems, such as $(\min ,+)$-convolution [18, 25], $(\min ,\max )$-convolution [13], min-witness convolution [26], convolution-3SUM [14] or even skew-convolution [12] cannot be easily adapted to $f$-Convolution as the hardness of these problems comes primarily from the ring operations.

The Orthogonal Vectors problem is related to the $f$-Query problem. In the Orthogonal Vectors problem we are given two sets of *n* vectors $A,B \subseteq \{0,1\}^d$ and the task is to decide if there is a pair $a \in A$, $b \in B$ such that $a \cdot b = 0$. In [38] it was shown that there is no algorithm with a running time of $n^{2-\epsilon } \cdot 2^{o(d)}$ for the Orthogonal Vectors problem for any $\epsilon > 0$, assuming SETH [36]. The currently best algorithm for Orthogonal Vectors runs in time $n^{2-1/{\mathcal {O}}(\log (d)/\log (n))}$ [1, 15], ${\mathcal {O}}(n \cdot 2^{cd})$ for some constant $c < 0.5$ [30], or ${\mathcal {O}}(|{\downarrow }A| + |{\downarrow }B|)$ [7] (where $|{\downarrow }F|$ is the total number of vectors whose support is a subset of the support of input vectors).

### Organization

In Sect. 2 we provide the formal definitions of the problems alongside the general statements of our results. In Sect. 3 we give an algorithm for $f$-Convolution that uses a given cyclic partition. In Sect. 4 we show that for every function $f :D\times D\rightarrow D$ there exists a cyclic partition of low cost. Finally, in Sect. 5 we give an algorithm for $f$-Query and prove Theorem 1.4. In Sect. 6 we conclude the paper and discuss future work.

## Preliminaries

Throughout the paper, we use Iverson bracket notation, where for the logic expression *P*, the value of $\llbracket {P}\rrbracket$ is 1 when *P* is true and 0 otherwise. For $n \in {\mathbb {N}}$ we use [*n*] to denote $\{1,\ldots ,n\}$. Through the paper we denote vectors in bold, for example, $\textbf{q}\in {\mathbb {Z}}^k$ denotes a *k*-dimensional vector of integers. We use subscripts to denote the entries of the vectors, e.g., $\textbf{q}{:}{=}(\textbf{q}_1,\ldots ,\textbf{q}_k)$.

Let *L*, *R* and *T* be arbitrary sets and let $f :L \times R \rightarrow T$ be an arbitrary function. We extend the definition of such an arbitrary function *f* to vectors as follows. For two vectors $\textbf{u}\in L^n$ and $\textbf{w}\in R^n$ we define

$$\begin{aligned} \textbf{u}\oplus _f \textbf{w}{:}{=}(f(\textbf{u}_1,\textbf{w}_1),\ldots , f(\textbf{u}_n,\textbf{w}_n)). \end{aligned}$$

In this paper, we consider the $f$-Convolution problem with a more general domain and image. We define it formally as follows:

### Definition 2.1

(*f*-Convolution) Let *L*, *R* and *T* be arbitrary sets and let $f:L \times R \rightarrow T$ be an arbitrary function. The $f$-Convolution of two functions $g:L^n \rightarrow {\mathbb {Z}}$ and $h:R^n\rightarrow {\mathbb {Z}}$, where $n\in {\mathbb {N}}$, is the function $(g \mathbin {\circledast _{f}}h):T^n \rightarrow {\mathbb {Z}}$ defined by

$$\begin{aligned} (g \mathbin {\circledast _{f}}h)(\textbf{v}) {:}{=}\sum _{\textbf{u}\in L^n,~\textbf{w}\in R^n} \llbracket { \textbf{v}= \textbf{u}\oplus _f \textbf{w}}\rrbracket \cdot g(\textbf{u}) \cdot h(\textbf{w}) \end{aligned}$$

for every $$\textbf{v}\in T^n$$.

As before the operations are taken in the standard ${\mathbb {Z}}(+,\cdot )$ ring and *M* is the maximum absolute value of the integers given on the input.

Now, we formally define the input and output to the $f$-Convolution problem.

### Definition 2.2

(*f*-Convolution Problem ($f$-Convolution)) Let *L*, *R* and *T* be arbitrary finite sets and let $f:L \times R \rightarrow T$ be an arbitrary function. The *f*-Convolution Problem is the following.

***Input:*** Two functions $g:R^n\rightarrow {\{-M,\ldots , M\}}$ and $h:L^n\rightarrow {\{-M,\ldots , M\}}$.

***Task:*** Compute $$g \mathbin {\circledast _{f}}h$$.

Our main result stated in the most general form is the following.

### Theorem 2.3

Let $f:L\times R\rightarrow T$ such that *L*, *R* and *T* are finite. There is an algorithm for the $f$-Convolution problem with $\widetilde{{\mathcal {O}}}(c^n \cdot \textrm{polylog}(M))$ time, where

$$\begin{aligned} c {:}{=}{\left\{ \begin{array}{ll} \frac{|L|}{2} \cdot \left( \vert {R} \vert + \frac{|T|}{2}\right) &{} \text {if } \vert {L} \vert \text { is even} \\ \frac{|L|-1}{2} \cdot \left( \vert {R} \vert + \frac{|T|}{2}\right) +|R| &{} \text {otherwise.} \end{array}\right. } \end{aligned}$$

Theorem 1.1 is a corollary of Theorem 2.3 by setting $L = R = T = D$.

The proof of Theorem 2.3 utilizes the notion of *cyclic partition*. For any $k\in {\mathbb {N}}$, let ${\mathbb {Z}}_k=\{0,1,\ldots , k-1\}$. We say a function $f:A\times B\rightarrow C$ is *k*-*cyclic* if, up to a relabeling of the sets *A*, *B* and *C*, it is an addition modulo *k*. Formally, $f:A\times B\rightarrow C$ is *k*-*cyclic* if there are $\sigma _A:A\rightarrow {\mathbb {Z}}_k$, $\sigma _B:B\rightarrow {\mathbb {Z}}_k$, and $\sigma _C :{\mathbb {Z}}_k \rightarrow C$ such that

$$\begin{aligned} \forall a\in A, ~b\in B:~~~f(a,b) = \sigma _C\left( \sigma _A(a)+ \sigma _B(b)\mod k\right) . \end{aligned}$$

We refer to the functions $\sigma _A$, $\sigma _B$ and $\sigma _C$ as the *relabeling* functions of *f*. For example, a constant function $f:A \times B \rightarrow \{0\}$ defined by $f(a,b)=0$ for all $(a,b)\in A\times B$ is 1-cyclic.

The *restriction* of $f:L\times R \rightarrow T$ to $A\subseteq L$ and $B\subseteq R$ is the function $g:A\times B\rightarrow T$ defined by $g(a,b) = f(a,b)$ for all $a\in A$ and $b\in B$. We say (*A*, *B*, *k*) is a *cyclic minor* of $f:L\times R \rightarrow T$ if the restriction of *f* to *A* and *B* is a *k*-cyclic function.

A *cyclic partition* of $f:L\times R \rightarrow T$ is a set of minors ${\mathcal {P}}=\{(A_1,B_1,k_1),\ldots , (A_m,B_m,k_m)\}$ such that $(A_i,B_i,k_i)$ is a cyclic minor of *f* and for every $(a,b)\in L\times R$ there is a unique $1\le i\le m$ such that $(a,b)\in A_i\times B_i$. The cost of the cyclic partition is $\textrm{cost}({\mathcal {P}})=\sum _{i=1}^{m} k_i$.

Theorem 2.3 follows from the following lemmas.

### Lemma 3.1

(Algorithm for Generalized Convolution) Let *L*, *R* and *T* be finite sets. Also, let $f:L\times R\rightarrow T$ be a function and let ${\mathcal {P}}$ be a cyclic partition of *f*. Then there is an $\widetilde{{\mathcal {O}}}((\textrm{cost}({\mathcal {P}})^n + |L|^n + |R|^n + |T|^n) \cdot \textrm{polylog}(M))$ time algorithm for $f$-Convolution.

### Lemma 4.1

Let $f:L\times R\rightarrow T$ where *L*, *R* and *T* are finite sets. Then there is a cyclic partition ${\mathcal {P}}$ of *f* such that $\textrm{cost}({\mathcal {P}})\le \frac{\vert {L} \vert }{2} \cdot (\vert {R} \vert + \frac{\vert {T} \vert }{2})$ when $\vert {L} \vert$ is even, and $\textrm{cost}({\mathcal {P}}) \le \vert {R} \vert + \frac{\vert {L} \vert -1}{2} \cdot (\vert {R} \vert + \frac{\vert {T} \vert }{2})$ when $\vert {L} \vert$ is odd.

The proof of Lemma 3.1 is included in Sect. 3 and proof of Lemma 4.1 is included in Sect. 4. The proof of Lemma 3.1 uses an algorithm for Cyclic Convolution.

### Definition 2.4

(Cyclic Convolution) Let $k\in {\mathbb {N}}$ and $\textbf{r}\in {\mathbb {N}}^k$. Also, let $g,h:Z\rightarrow {\mathbb {N}}$ be two functions where $Z={\mathbb {Z}}_{\textbf{r}_1}\times \cdots \times {\mathbb {Z}}_{\textbf{r}_k}$. The Cyclic Convolution of *g* and *h* is the function $(g\mathbin {\odot }h):Z \rightarrow {\mathbb {N}}$ defined by

$$\begin{aligned} (g\mathbin {\odot }h)(\textbf{v}) = \sum _{\textbf{u},\textbf{w}\in Z} \left( \prod _{i=1}^k \llbracket {\textbf{u}_i + \textbf{w}_i = \textbf{v}_i \mod \textbf{r}_i}\rrbracket \right) \cdot g(\textbf{u})\cdot h(\textbf{w}) \end{aligned}$$

for every $$\textbf{v}\in Z$$.

For any $K\subseteq {\mathbb {N}}$ we define the *K*-${\textsc {Cyclic Convolution Problem}}$ in which we restrict the entries of the vector $\textbf{r}$ in Definition 2.4 to be in *K*.

### Definition 2.5

(*K*-${\textsc {Cyclic Convolution Problem}}$) For any $K\subseteq {\mathbb {N}}$ the *K*-Cyclic Convolution Problem is defined as follows.

***Input:*** Integers $k, M \in {\mathbb {N}}$, a vector $\textbf{r}\in {\mathbb {N}}^k$ such that $\textbf{r}_j \in K$ for every $j\in [k]$ and two functions $g,h:Z\rightarrow {\{-M,\ldots , M\}}$ where $Z={\mathbb {Z}}_{\textbf{r}_1}\times \cdots \times {\mathbb {Z}}_{\textbf{r}_k}$.

***Task:*** Compute the ${\textsc {Cyclic Convolution}}$ $g\mathbin {\odot }h :Z \rightarrow {\mathbb {Z}}$.

Van Rooij [33] claimed that the ${\mathbb {N}}$-Cyclic Convolution Problem can be solved in $\widetilde{{\mathcal {O}}}\left( \big (\prod _{i=1}^k \textbf{r}_i\big ) \cdot \textrm{polylog}(M)\right)$ time. However, for his algorithm to work it must be given an appropriate large prime *p* and several primitives roots of unity in ${{\mathbb {F}}}_p$. We are unaware of a method which deterministically finds such a prime and roots while retaining the running time. To overcome this obstacle we present an algorithm for the *K*-Cyclic Convolution Problem when $K\subseteq {\mathbb {N}}$ is a fixed finite set. Our solution uses multiple smaller primes and the Chinese Reminder Theorem. We include the details in Appendix A.

### Theorem 2.6

(*K*-Cyclic Convolution) For any finite set $K\subseteq {\mathbb {N}}$, there is an $\widetilde{{\mathcal {O}}}\left( (\prod _{i=1}^k \textbf{r}_i) \cdot \textrm{polylog}(M)\right)$ algorithm for the *K*-Cyclic Convolution Problem.

## Generalized Convolution

In this section we prove Lemma 3.1.

### Lemma 3.1

(Algorithm for Generalized Convolution) Let *L*, *R* and *T* be finite sets. Also, let $f:L\times R\rightarrow T$ be a function and let ${\mathcal {P}}$ be a cyclic partition of *f*. Then there is an $\widetilde{{\mathcal {O}}}((\textrm{cost}({\mathcal {P}})^n + |L|^n + |R|^n + |T|^n) \cdot \textrm{polylog}(M))$ time algorithm for $f$-Convolution.

Throughout the section we fix *L*, *R* and *T*, and $f :L \times R \rightarrow T$ to be as in the statement of Lemma 3.1. Additionally, fix a cyclic partition ${\mathcal {P}}= \{(A_1, B_1, k_1), \ldots , (A_m, B_m, k_m)\}$. Furthermore, let $\sigma _{A,i}$, $\sigma _{B,i}$ and $\sigma _{C,i}$ be the relabeling functions of the cyclic minor $(A_i,B_i,k_i)$ for every $i \in [m]$. We assume the labeling functions are also fixed throughout this section.

In order to describe our algorithm for Lemma 3.1, we first need to establish several technical definitions.

### Definition 3.2

(Type) The *type* of two vectors $\textbf{u}\in L^{n}$ and $\textbf{w}\in R^{n}$ is the unique vector ${\varvec{p}}\in [m]^{n}$ for which $\textbf{u}_i \in A_{{\varvec{p}}_i}$ and $\textbf{w}_i \in B_{{\varvec{p}}_i}$ for all $i \in [n]$.

Observe that the type of two vectors is well defined as ${\mathcal {P}}$ is a cyclic partition. For any type ${\varvec{p}}\in \{1, \ldots , m\}^{n}$ we define

$$\begin{aligned} L_{{\varvec{p}}}{:}{=}A_{{\varvec{p}}_1} \times \dots \times A_{{\varvec{p}}_n},{} & {} R_{{\varvec{p}}}{:}{=}B_{{\varvec{p}}_1} \times \dots \times B_{{\varvec{p}}_n},{} & {} Z_{{\varvec{p}}}{:}{=}{\mathbb {Z}}_{k_{{\varvec{p}}_1}} \times \dots \times {\mathbb {Z}}_{k_{{\varvec{p}}_n}} \end{aligned}$$

to be vector domains restricted to type ${\varvec{p}}$. For any type ${\varvec{p}}$ we introduce relabeling functions on its restricted domains. The relabeling functions of ${\varvec{p}}$ are the functions $\varvec{\sigma }_{{\varvec{p}}}^{L}:L_{{\varvec{p}}}\rightarrow Z_{{\varvec{p}}}$, $\varvec{\sigma }_{{\varvec{p}}}^{R}:R_{{\varvec{p}}}\rightarrow Z_{{\varvec{p}}}$, and $\varvec{\sigma }_{{\varvec{p}}}^{T}:Z_{{\varvec{p}}}\rightarrow T^{n}$ defined as follows:

$$\begin{aligned} \varvec{\sigma }_{{\varvec{p}}}^{L}(\textbf{v})&{:}{=}\left( \sigma _{A, {\varvec{p}}_1}(\textbf{v}_1), \ldots , \sigma _{A, {\varvec{p}}_n}(\textbf{v}_n) \right)&\forall \textbf{v}\in L_{{\varvec{p}}},\\ \varvec{\sigma }_{{\varvec{p}}}^{R}(\textbf{v})&{:}{=}\left( \sigma _{B, {\varvec{p}}_1}(\textbf{v}_1), \ldots , \sigma _{B, {\varvec{p}}_n}(\textbf{v}_n) \right)&\forall \textbf{v}\in R_{{\varvec{p}}},\\ \varvec{\sigma }_{{\varvec{p}}}^{T}(\textbf{q})&{:}{=}\left( \sigma _{C, {\varvec{p}}_1}(\textbf{q}_1), \ldots , \sigma _{C, {\varvec{p}}_n}(\textbf{q}_n) \right)&\forall \textbf{q}\in Z_{{\varvec{p}}}. \end{aligned}$$

Our algorithm heavily depends on constructing the following projections.

### Definition 3.3

(Projection of function) The projection of a function $g:L^{n} \rightarrow {\mathbb {Z}}$ with respect to the type ${\varvec{p}}\in [m]^{n}$, is the function $g_{\varvec{p}}:Z_{{\varvec{p}}}\rightarrow {\mathbb {Z}}$ defined as

$$\begin{aligned} g_{{\varvec{p}}}(\textbf{q}) {:}{=}\sum \limits _{\textbf{u}\in L_{{\varvec{p}}}}\llbracket { \varvec{\sigma }_{{\varvec{p}}}^{L}(\textbf{u}) = \textbf{q}}\rrbracket \cdot g(\textbf{u}){} & {} \text { for every } \textbf{q}\in Z_{{\varvec{p}}}. \end{aligned}$$

Similarly, the projection $h_{\varvec{p}}:Z_{{\varvec{p}}}\rightarrow {\mathbb {Z}}$ of a function $h :R^{n} \rightarrow {\mathbb {Z}}$ with respect to the type ${\varvec{p}}\in [m]^n$ is defined as

$$\begin{aligned} h_{{\varvec{p}}}(\textbf{q}) {:}{=}\sum \limits _{\textbf{w}\in R_{{\varvec{p}}}}\llbracket { \varvec{\sigma }_{{\varvec{p}}}^{R}(\textbf{w}) = \textbf{q}}\rrbracket \cdot h(\textbf{w}){} & {} \text { for every } \textbf{q}\in Z_{{\varvec{p}}}. \end{aligned}$$

The projections are useful due to the following connection with $g\mathbin {\circledast _{f}}h$.

### Lemma 3.4

Let $g :L^{n} \rightarrow {\mathbb {Z}}$ and $h :R^{n} \rightarrow {\mathbb {Z}}$, then for every $\textbf{v}\in T^n$ it holds that:

$$\begin{aligned} \left( g \mathbin {\circledast _{f}}h\right) (\textbf{v}) = \sum _{{\varvec{p}}\in [m]^{n}} \sum _{\textbf{q}\in Z_{{\varvec{p}}}} \llbracket {\varvec{\sigma }_{{\varvec{p}}}^{T}(\textbf{q}) = \textbf{v}}\rrbracket \cdot \left( g_{\varvec{p}}\mathbin {\odot }h_{\varvec{p}}\right) (\textbf{q}) , \end{aligned}$$

where $g_{\varvec{p}}\mathbin {\odot }h_{\varvec{p}}$ is the cyclic convolution of $g_{\varvec{p}}$ and $h_{\varvec{p}}$.

We give the proof of Lemma 3.4 in Sect. 3.1. It should be noted that the naive computation of the projection functions of *g* and *h* with respect to all types ${\varvec{p}}$ is significantly slower than the running time stated in Lemma 3.1. To adhere to the stated running time we use a dynamic programming procedure for the computations, as stated in the following lemma.

### Lemma 3.5

There exists an algorithm which given a function $g:L^n \rightarrow {\{-M,\ldots , M\}}$ returns the set of its projections, $\{g_{\varvec{p}}\mid {\varvec{p}}\in [m]^{n} \}$, in time $\left( \left( \textrm{cost}({\mathcal {P}})^n + |L|^n\right) \right)$.

### Remark 3.6

Analogously, we can also construct every projection of a function $h :R^n \rightarrow {\{-M,\ldots , M\}}$ in $\widetilde{{\mathcal {O}}}\left( \left( \textrm{cost}({\mathcal {P}})^n + |R|^n\right) \cdot \textrm{polylog}(M)\right)$ time.

The proof of Lemma 3.5 in given in Sect. 3.1.

Our algorithm for $f$-Convolution (see Algorithm 1 for the pseudocode) is a direct implication of Lemmas 3.4 and 3.5. First, the algorithm computes the projections of *g* and *h* with respect to every type ${\varvec{p}}$. Subsequently, the cyclic convolution of $g_{\varvec{p}}$ and $h_{\varvec{p}}$ is computed efficiently as described in Theorem 2.6. Finally, the values of $(g \mathbin {\circledast _{f}}h)$ are reconstructed by the formula in Lemma 3.4.

**Algorithm 1:**
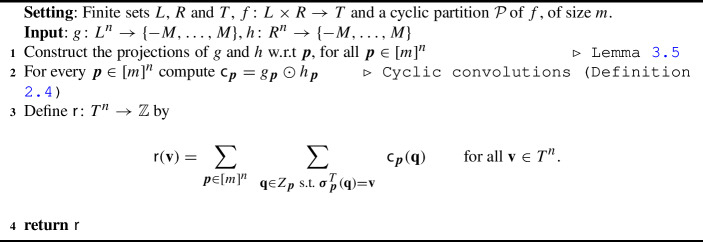
Cyclic Partition Algorithm for the $f$-Convolution problem

### Proof of Lemma 3.1

Observe that Algorithm 1 returns ${\textsf{r}} :T^n\rightarrow {\mathbb {Z}}$ such that for every $\textbf{v}\in T^n$ it holds that

$$\begin{aligned} {\textsf{r}}(\textbf{v}) = \sum _{{\varvec{p}}\in [m]^{n}} \sum _{\begin{array}{c} \textbf{q}\in Z_{{\varvec{p}}}\\ \text {s.t. } \varvec{\sigma }_{{\varvec{p}}}^{T}(\textbf{q}) = \textbf{v} \end{array}} {\textsf{c}}_{{\varvec{p}}}(\textbf{q}) = \sum _{{\varvec{p}}\in [m]^{n}} \sum _{\textbf{q}\in Z_{{\varvec{p}}}} \llbracket {\varvec{\sigma }_{{\varvec{p}}}^{T}(\textbf{q}) = \textbf{v}}\rrbracket \cdot \left( g_{\varvec{p}}\mathbin {\odot }h_{\varvec{p}}\right) (\textbf{q})=\left( g \mathbin {\circledast _{f}}h\right) (\textbf{v}), \end{aligned}$$

where the last equality is by Lemma 3.4. Thus, the algorithm returns $(g\mathbin {\circledast _{f}}h)$ as required. It therefore remains to bound the running time of the algorithm.

By Lemma 3.5, Line 1 of Algorithm 1 runs in time $\widetilde{{\mathcal {O}}}((\textrm{cost}({\mathcal {P}})^n + |L|^n+|R|^n) \cdot \textrm{polylog}(M))$. Define $K=\{ k \mid (A,B,k) \in {\mathcal {P}}\}=\{k_1,\ldots , k_m\}$ be different costs of cyclic minors in ${\mathcal {P}}$. By Theorom 2.6, for any type ${\varvec{p}}\in [m]^n$ the computation of $g_{\varvec{p}}\mathbin {\odot }h_{{\varvec{p}}}$ in Line 2 is an instance of *K*-Cyclic Convolution Problem which can be solved in time $\widetilde{{\mathcal {O}}}((\prod _{ i=1}^{n} k_{{\varvec{p}}_i}) \cdot \textrm{polylog}(M))$. Thus the overall running time of Line 2 is $\widetilde{{\mathcal {O}}}\left( (\sum _{{\varvec{p}}\in [m]^{n} } \prod _{i=1}^n k_{{\varvec{p}}_i}) \cdot \textrm{polylog}(M)\right)$.

Finally, observe that the construction of ${\textsf{r}}$ in Line 3 can be implemented by initializing ${\textsf{r}}$ to be zeros and iteratively adding the value of ${\textsf{c}}_{{\varvec{p}}}(\textbf{q})$ to ${\textsf{r}}(\sigma ^T_{{\varvec{p}}}(\textbf{q}))$ for every ${\varvec{p}}\in [m]^n$ and $\textbf{q}\in Z_{{\varvec{p}}}$. The required running time is thus $\widetilde{{\mathcal {O}}}(|T|^n \cdot \textrm{polylog}(M))$ for the initialization and $\widetilde{{\mathcal {O}}}\left( (\sum _{{\varvec{p}}\in [m]^n} |Z_{{\varvec{p}}}|) \cdot \textrm{polylog}(M)\right) =\left( (\sum _{{\varvec{p}}\in [m]^n} \prod _{i=1}^{n} k_{{\varvec{p}}_i})\right)$ for the addition operations. Thus, the overall running time of Line 3 is

$$\begin{aligned} \widetilde{{\mathcal {O}}}\left( \left( |T|^n+ \sum _{{\varvec{p}}\in [m]^{n} } \prod _{i=1}^n k_{{\varvec{p}}_i}\right) \cdot \textrm{polylog}(M)\right) . \end{aligned}$$

Combining the above, with $\sum _{{\varvec{p}}\in [m]^{n} } \prod _{i=1}^n k_{{\varvec{p}}_i}= \left( \sum _{i=1}^{m} k_i \right) ^n = \left( \textrm{cost}({\mathcal {P}})\right) ^n$ means that the running time of Algorithm 1 is

$$\begin{aligned} \widetilde{{\mathcal {O}}}\left( \left( |T|^n + |R|^n + |L|^n+\textrm{cost}({\mathcal {P}})^n\right) \cdot \textrm{polylog}(M)\right) \end{aligned}$$

This concludes the proof of Lemma 3.1. $\square$

### Properties of Projections

In this section we provide the proofs for Lemmas 3.4 and 3.5. The proof of Lemma 3.4 uses the following definitions of coordinate-wise addition with respect to a type ${\varvec{p}}$.

#### Definition 3.7

(Coordinate-wise addition modulo for type) For any ${\varvec{p}}\in [m]^n$ we define a coordinate-wise addition modulo as

$$\begin{aligned} \textbf{q}+_{\varvec{p}}\textbf{r}{:}{=}\left( (\textbf{q}_1 + \textbf{r}_1 \mod k_{{\varvec{p}}_1}),\ldots ,(\textbf{q}_n + \textbf{r}_n \mod k_{{\varvec{p}}_n}) \right){} & {} \text { for every } \textbf{q},\textbf{r}\in Z_{{\varvec{p}}}. \end{aligned}$$

#### Proof of Lemma 3.4

By Definition 2.1 it holds that:

3.1 $$\begin{aligned} \left( g \mathbin {\circledast _{f}}h\right) (\textbf{v}) = \sum _{\textbf{u}\in L^{n}, \textbf{w}\in R^{n}} \llbracket {\textbf{v}= \textbf{u}\oplus _f \textbf{w}}\rrbracket \cdot g(\textbf{u}) \cdot h(\textbf{w}). \end{aligned}$$

Recall that the type of every two vectors $(\textbf{u},\textbf{w})\in L^n \times R^n$ is unique and $[m]^n$ contains all possible types and hence, we can rewrite (3.1) as

3.2 $$\begin{aligned} ( g \mathbin {\circledast _{f}}h)(\textbf{v})&= \sum _{{\varvec{p}}\in [m]^n} \sum _{\textbf{u}\in L_{{\varvec{p}}}, \textbf{w}\in R_{{\varvec{p}}}} g(\textbf{u})\cdot h(\textbf{w}) \cdot \llbracket {\textbf{v}= \textbf{u}\oplus _f\textbf{w}}\rrbracket \end{aligned}$$

By the properties of the relabeling functions, we get

$$\begin{aligned}&=\sum _{{\varvec{p}}\in [m]^{n}} \sum _{\textbf{u}\in L_{{\varvec{p}}}, \textbf{w}\in R_{{\varvec{p}}}} g(\textbf{u}) \cdot h(\textbf{w}) \cdot \llbracket {\textbf{v}= \varvec{\sigma }_{{\varvec{p}}}^{T}\left( \varvec{\sigma }_{{\varvec{p}}}^{L}(\textbf{u}) +_{\varvec{p}}\varvec{\sigma }_{{\varvec{p}}}^{R}(\textbf{w}) \right) }\rrbracket \nonumber \\&= \sum _{{\varvec{p}}\in [m]^{n}} \sum _{\textbf{q}\in Z_{{\varvec{p}}}} \sum _{\textbf{u}\in L_{{\varvec{p}}}, \textbf{w}\in R_{{\varvec{p}}}} g(\textbf{u}) \cdot h(\textbf{w}) \cdot \llbracket {\textbf{v}= \varvec{\sigma }_{{\varvec{p}}}^{T}(\textbf{q})}\rrbracket \cdot \llbracket {\textbf{q}= \varvec{\sigma }_{{\varvec{p}}}^{L}(\textbf{u}) +_{\varvec{p}}\varvec{\sigma }_{{\varvec{p}}}^{R}(\textbf{w})}\rrbracket \nonumber \\&= \sum _{{\varvec{p}}\in [m]^{n}} \sum _{\begin{array}{c} \textbf{q}\in Z_{{\varvec{p}}}\nonumber \\ \text { s.t. } \varvec{\sigma }_{{\varvec{p}}}^{T}(\textbf{q}) = \textbf{v} \end{array}} \sum _{~\textbf{u}\in L_{{\varvec{p}}}, \textbf{w}\in R_{{\varvec{p}}}~} g(\textbf{u}) \cdot h(\textbf{w}) \cdot \llbracket {\textbf{q}= \varvec{\sigma }_{{\varvec{p}}}^{L}(\textbf{u}) +_{\varvec{p}}\varvec{\sigma }_{{\varvec{p}}}^{R}(\textbf{w})}\rrbracket . \end{aligned}$$

Observe that we can *partition* $L_{{\varvec{p}}}$ (respectively $R_{{\varvec{p}}}$) by considering the inverse images of $\textbf{r}\in Z_{{\varvec{p}}}$ under $\varvec{\sigma }_{{\varvec{p}}}^{L}$ (respectively $\varvec{\sigma }_{{\varvec{p}}}^{R}$), i.e. $L_{{\varvec{p}}}= \biguplus _{\textbf{r}\in Z_{{\varvec{p}}}} \{\textbf{u}\in L_{{\varvec{p}}}\mid \varvec{\sigma }_{{\varvec{p}}}^{L}(\textbf{u}) = \textbf{r}\}$. Hence, for every ${\varvec{p}}\in [m]^n$ and $\textbf{q}\in Z_{{\varvec{p}}}$ it holds that

3.3 $$\begin{aligned}&\sum _{~\textbf{u}\in L_{{\varvec{p}}}, \textbf{v}\in R_{{\varvec{p}}}~} g(\textbf{u}) \cdot h(\textbf{w}) \cdot \llbracket {\textbf{q}= \varvec{\sigma }_{{\varvec{p}}}^{L}(\textbf{u}) +_{\varvec{p}}\varvec{\sigma }_{{\varvec{p}}}^{R}(\textbf{w})}\rrbracket \nonumber \\&\quad = \sum _{\textbf{r}, \textbf{s}\in Z_{{\varvec{p}}}} \sum _{~\textbf{u}\in L_{{\varvec{p}}}, \textbf{w}\in R_{{\varvec{p}}}~} g(\textbf{u}) \cdot h(\textbf{w}) \cdot \llbracket {\textbf{q}= \textbf{r}+_{\varvec{p}}\textbf{s}}\rrbracket \cdot \llbracket {\textbf{r}= \varvec{\sigma }_{{\varvec{p}}}^{L}(\textbf{u})}\rrbracket \cdot \llbracket {\textbf{s}=\varvec{\sigma }_{{\varvec{p}}}^{R}(\textbf{w})}\rrbracket \nonumber \\&\quad =\sum _{\textbf{r}, \textbf{s}\in Z_{{\varvec{p}}}} \llbracket {\textbf{q}= \textbf{r}+_{\varvec{p}}\textbf{s}}\rrbracket \left( \sum _{\textbf{u}\in L_{{\varvec{p}}}}\llbracket {\textbf{r}= \varvec{\sigma }_{{\varvec{p}}}^{L}(\textbf{u})}\rrbracket \cdot g(\textbf{u}) \right) \cdot \left( \sum _{\textbf{w}\in R_{{\varvec{p}}}} \llbracket {\textbf{s}=\varvec{\sigma }_{{\varvec{p}}}^{R}(\textbf{w})}\rrbracket \cdot h(\textbf{w})\right) \nonumber \\&\quad =\sum _{\textbf{r}, \textbf{s}\in Z_{{\varvec{p}}}} \llbracket {\textbf{q}= \textbf{r}+_{\varvec{p}}\textbf{s}}\rrbracket \cdot g_{{\varvec{p}}}(\textbf{r})\cdot h_{{\varvec{p}}} (\textbf{s})\nonumber \\&\quad = \;(g_{{\varvec{p}}} \mathbin {\odot }h_{{\varvec{p}}})(\textbf{q}). \end{aligned}$$

By plugging (3.3) into (3.2) we get

$$\begin{aligned} \left( g \mathbin {\circledast _{f}}h\right) (\textbf{v})&= \sum _{{\varvec{p}}\in [m]^{n}} \sum _{\begin{array}{c} \textbf{q}\in Z_{{\varvec{p}}}\\ \text { s.t. } \varvec{\sigma }_{{\varvec{p}}}^{T}(\textbf{q}) = \textbf{v} \end{array}} (g_{{\varvec{p}}}\mathbin {\odot }h_{{\varvec{p}}})(\textbf{q})\\ {}&= \sum _{{\varvec{p}}\in [m]^{n}} \sum _{\textbf{q}\in Z_{{\varvec{p}}}} \llbracket {\varvec{\sigma }_{{\varvec{p}}}^{T}(\textbf{q}) = \textbf{v}}\rrbracket \cdot \left( g_{\varvec{p}}\mathbin {\odot }h_{\varvec{p}}\right) (\textbf{q}), \end{aligned}$$

as required. $$\square $$

#### Proof of Lemma 3.5

The idea is to use a dynamic programming algorithm loosely inspired by Yates’s algorithm [40].

Define $X^{(\ell )} =\left\{ ({\varvec{p}}, \textbf{q})~\big |~{\varvec{p}}\in [m]^{\ell },~\textbf{q}\in {\mathbb {Z}}_{{\varvec{p}}_1} \times \dots \times {\mathbb {Z}}_{{\varvec{p}}_\ell }\right\}$ for every $\ell \in \{0,\ldots , n\}$. We use $X^{(\ell )}$ to define a dynamic programming table ${\textsf{DP}}^{(\ell )}:X^{(\ell )} \times L^{n-\ell } \rightarrow {\mathbb {Z}}$ for every $\ell \in \{0,\ldots n\}$ by:

$$\begin{aligned}&{\textsf{DP}}^{(\ell )}[({\varvec{p}}_1, \ldots , {\varvec{p}}_\ell ),(\textbf{q}_1,\ldots ,\textbf{q}_\ell )][\textbf{t}_{\ell +1},\ldots ,\textbf{t}_{n}]\\&{:}{=}\sum _{\begin{array}{c} \textbf{t}_1 \in A_{{\varvec{p}}_1}\\ \ldots \\ \textbf{t}_\ell \in A_{{\varvec{p}}_\ell } \end{array}} \left( \prod _{i=1}^\ell \llbracket {\sigma _{{\varvec{p}}_i}(\textbf{t}_i) = \textbf{q}_i}\rrbracket \right) \cdot g(\textbf{t}_1, \ldots , \textbf{t}_n) . \end{aligned}$$

The tables ${\textsf{DP}}^{(0)},{\textsf{DP}}^{(1)},\ldots , {\textsf{DP}}^{(n)}$ are computed consecutively where the computation of ${\textsf{DP}}^{(\ell )}$ relies on the values of ${\textsf{DP}}^{(\ell -1)}$ for any $\ell \in [n]$. Observe that $g_{\varvec{p}}(\textbf{q}) = {\textsf{DP}}^{(n)}[({\varvec{p}}_1,\ldots ,{\varvec{p}}_n),(\textbf{q}_1,\ldots ,\textbf{q}_n)][\varepsilon ]$ for every ${\varvec{p}}$ and $\textbf{q}$, which means that computing ${\textsf{DP}}^{(n)}$ is equivalent to computing the projection functions $g_{{\varvec{p}}}$ of *g* for every type ${\varvec{p}}$.^5^

It holds that ${\textsf{DP}}^{(0)}[\varepsilon ,\varepsilon ][\textbf{t}] = g(\textbf{t})$. Hence, ${\textsf{DP}}^{(0)}$ can be trivially computed in $|L|^n$ time. We use the following straightforward recurrence to compute ${\textsf{DP}}^{(\ell )}$:

3.4 $$\begin{aligned} \begin{aligned}&{\textsf{DP}}^{(\ell )} [({\varvec{p}}_1, \ldots , {\varvec{p}}_\ell ),(\textbf{q}_1,\ldots ,\textbf{q}_\ell )][\textbf{t}_{\ell +1},\ldots ,\textbf{t}_{n}] \\&\qquad = \sum _{\textbf{t}_\ell \in A_{{\varvec{p}}_\ell }} \llbracket {\sigma _{{\varvec{p}}_\ell }(\textbf{t}_\ell ) = \textbf{q}_\ell }\rrbracket \cdot {\textsf{DP}}^{(\ell - 1)}[({\varvec{p}}_1, \ldots , {\varvec{p}}_{\ell -1}),(\textbf{q}_1,\ldots ,\textbf{q}_{\ell -1})][\textbf{t}_{\ell },\ldots ,\textbf{t}_{n}]. \end{aligned} \end{aligned}$$

A dynamic programming algorithm which computes ${\textsf{DP}}^{(n)}$ can be easily derived from (3.4) and the formula for ${\textsf{DP}}^{(0)}$. The total number of states in the dynamic programming table ${\textsf{DP}}^{(\ell )}$ is

$$\begin{aligned} \left( \sum _{{\varvec{p}}\in [m]^{\ell }} \left( k_{{\varvec{p}}_1} \cdot \ldots \cdot k_{{\varvec{p}}_\ell }\right) \right) \cdot \vert {L} \vert ^{n-\ell } = \left( k_1 + \dots + k_m \right) ^{\ell } \cdot \vert {L} \vert ^{n-\ell }&= \textrm{cost}({\mathcal {P}})^{\ell } \cdot \vert {L} \vert ^{n-\ell }. \end{aligned}$$

This is bounded by $\textrm{cost}({\mathcal {P}})^n + |L|^n$ for every $\ell \in [n]$. To transition between states we spend polynomial time per entry because we assume that $|L| = {\mathcal {O}}(1)$. Hence, we can compute $g_{\varvec{p}}$ for every ${\varvec{p}}$ in $\widetilde{{\mathcal {O}}}((\textrm{cost}({\mathcal {P}})^n + |L|^n) \cdot \textrm{polylog}(M))$ time. $\square$

## The Existence of a Low-Cost Cyclic Partition

In this section we prove Lemma 4.1.

### Lemma 4.1

Let $f:L\times R\rightarrow T$ where *L*, *R* and *T* are finite sets. Then there is a cyclic partition ${\mathcal {P}}$ of *f* such that $\textrm{cost}({\mathcal {P}})\le \frac{\vert {L} \vert }{2} \cdot (\vert {R} \vert + \frac{\vert {T} \vert }{2})$ when $\vert {L} \vert$ is even, and $\textrm{cost}({\mathcal {P}}) \le \vert {R} \vert + \frac{\vert {L} \vert -1}{2} \cdot (\vert {R} \vert + \frac{\vert {T} \vert }{2})$ when $\vert {L} \vert$ is odd.

We first consider the special case when $\vert {L} \vert =2$. Later we reduce the general case to this scenario and use the result as a black-box.

As a warm-up we construct a cyclic partition of cost at most $\frac{7}{8} \vert {D} \vert ^2$ assuming that $L=R=T=D$ and that $\vert {D} \vert$ is even. For this, we first partition $D$ into pairs $d_1^{(i)},d_2^{(i)}$ where $i\in [\vert {D} \vert /2]$ and show for each such pair that *f* restricted to $\{d_1^{(i)},d_2^{(i)}\}$ and $D$ has a cyclic partition of cost at most $\frac{7}{4} \vert {D} \vert$. The union of these cyclic partitions forms a cyclic partition of *f* with cost at most $\frac{\vert {D} \vert }{2} \cdot \frac{7}{4} \vert {D} \vert = \frac{7}{8} \vert {D} \vert ^2$.

To construct the cyclic partition for a fixed $i\in [\vert {D} \vert /2]$, we find a maximal number *r* of pairwise disjoint pairs $e_1^{(j)},e_2^{(j)} \in D$ such that $\vert {\{f(d_{a}^{(i)},e_b^{(j)}) \mid a,b \in \{1,2\} \}} \vert \le 3$ for each $j \in [r]$, i.e. for each *j* at least one of the four values $f(d_1^{(i)},e_1^{(j)}),f(d_1^{(i)},e_2^{(j)}),f(d_2^{(i)},e_1^{(j)}),f(d_2^{(i)},e_2^{(j)})$ repeats. With this assumption, *f* restricted to $\{d_1^{(i)},d_2^{(i)}\}$ and $\{e_1^{(j)},e_2^{(j)}\}$ is either a cyclic minor of cost at most 3 or can be decomposed into 3 trivial cyclic minors of the total cost at most 3. We claim that $r \ge \vert {D} \vert /4$. Indeed, assume that there are fewer than |*D*|/4 such pairs, i.e. $r < \vert {D} \vert /4$. Let ${\overline{D}}$ denote the $\vert {D} \vert -2 \cdot r > \vert {D} \vert /2$ remaining values in $D$. As the set $\{f(d_a^{(i)}, d) \mid d \in {\overline{D}}, a \in \{1,2\} \}$ can only contain at most $\vert {D} \vert$ values, we can find another pair $e_1^{(r+1)},e_2^{(r+1)}$ with the above constraints. Note that *f* restricted to $\{d_1^{(i)},d_2^{(i)}\}$ and $\overline{D}$ can be decomposed into at most $2\vert {{\overline{D}}} \vert$ trivial minors. Hence, the cyclic partition for *f* restricted to $\{d_1^{(i)},d_2^{(i)}\}$ and $D$ has cost at most

$$\begin{aligned} 3 r + 2 \cdot \vert {{\overline{D}}} \vert \le 3 \cdot \frac{\vert {D} \vert }{4} + 2 \cdot \frac{\vert {D} \vert }{2} \le \frac{7}{4} \vert {D} \vert . \end{aligned}$$

### Special Case: $|L| = 2$

In this section, we prove the following lemma that is a special case of Lemma 4.1.

#### Lemma 4.2

If $f:L \times R \rightarrow T$ with $\vert {L} \vert =2$, then there is a cyclic partition ${\mathcal {P}}$ of *f* such that $\textrm{cost}({\mathcal {P}}) \le \vert {R} \vert + \vert {T} \vert /2$.

To construct the cyclic partition we proceed as follows. First, we define, for a function *f*, the representation graph $G_f$. Next, we show that if this graph has a special structure, which we later call *nice*, then we can easily find a cyclic partition for the function *f*. Afterwards we decompose (the edges of) an arbitrary representation graph $G_f$ into nice structures and then combine the cyclic partitions coming from these parts to a cyclic partition for the original function *f*.

#### Definition 4.3

(Graph Representation) Let $f:L \times R \rightarrow T$ be such that $\vert {L} \vert =2$ with $L = \{\ell _0, \ell _1\}$.

We say a function $\lambda _f:R \rightarrow T \times T$ with $\lambda _f:r \mapsto (f(\ell _0,r),f(\ell _1,r))$ is the *edge mapping* of *f*. We say that a directed graph $G_f$ (which might have self-loops) with vertex set $V(G_f) {:}{=}T$ and edge set $E(G_f) {:}{=}\{ \lambda _f(r) \mid r \in R\}$ is the *representation graph* of *f*.

We say that the representation graph $G_f$ is *nice* if $G_f$ is a directed cycle or a directed path (potentially with a single edge).

As a next step we define the restriction of a function based on a subgraph of the corresponding representation graph.

#### Definition 4.4

(Restriction of *f*) Let $f:L \times R \rightarrow T$ be a function such that $\vert {L} \vert =2$

and let $G_f$ be the representation graph of *f*. Let $E'\subseteq E(G_f)$ be a given subset of edges inducing the subgraph $G'$ of $G_f$.

Based on $E'$ (and thus, $G'$) we define a new function $f'$ in the following and say that $f'$ is the *function represented by* $G'$ or $E'$.

With $T'{:}{=}V(G')$ and $R'{:}{=}\{ r \in R \mid \lambda _f(r) \in E'\}$, we define $f' :L \times R' \rightarrow T'$ as the *restriction* of *f* such that the representation graph of $f'$ is $G'$. Formally, we set $f'(\ell ,r) {:}{=}f(\ell ,r)$ for all $\ell \in L$ and $r \in R'$.

A decomposition of a directed graph *G* is a family ${\mathcal {F}}$ of edge-disjoint subgraphs of *G*, such that each edge belongs to exactly one subgraph in ${\mathcal {F}}$. The following observation follows directly from the previous definition.

#### Observation 4.5

Let $\{G_1,\dots ,G_k\}$ be a decomposition of the graph $G_f$ into *k* subgraphs, let $f_i$ be the function represented by $G_i$, and let ${\mathcal {P}}_i$ be a cyclic partition of $f_i$.

Then ${\mathcal {P}}=\bigcup _{i\in [k]} {\mathcal {P}}_i$ is a cyclic partition of *f* with cost $\textrm{cost}({\mathcal {P}}) = \sum _{i\in [k]} \textrm{cost}({\mathcal {P}}_i)$.

**Fig. 2:**
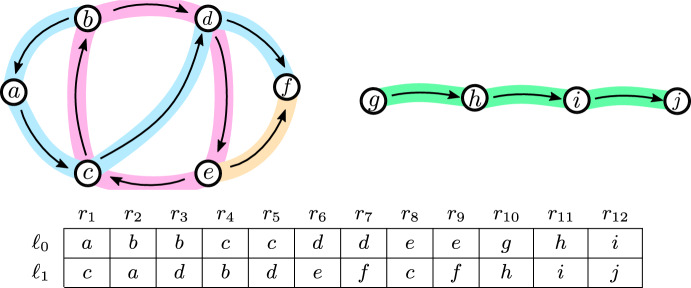
Example of the construction of a representation graph from the function *f* to obtain a cyclic partition. We put an edge between vertices *u* and *v* if there is an $r_i$ with $u = f(\ell _0,r_i)$ and $v = f(\ell _1,r_i)$. We highlight an example decomposition of the edges into a cycle with 4 vertices (highlighted red) and three paths with 5, 2 and 4 vertices (highlighted blue, yellow and green respectively). The cost of this cyclic partition is $4 + 5 + 2 + 4 = 15$ (Color figure online)

**Cyclic Partitions Using Nice Representation Graphs** As a next step, we show that functions admit cyclic partitions if the representation graph is nice. We extend these results to functions with arbitrary representation graphs by decomposing these graphs into nice subgraphs. Finally, we combine these results to obtain a cyclic partition for the original function *f* (Fig. 2).

#### Lemma 4.6

Let $f:L \times R \rightarrow T$ be a function such that $G_f$ is nice. Then *f* has a cyclic partition of cost at most $\vert {T} \vert =\vert {V(G_f)} \vert$.

#### Proof

By definition, a nice graph is either a cycle or a path. We handle each case separately in the following. Let $L = \{\ell _0, \ell _1\}$.

$$G_f$$ is a cycle.
We first define the relabeling functions of *f* to show that *f* is $\vert {T} \vert$-cyclic. For the elements in *L*, let $\sigma _L :L \rightarrow {\mathbb {Z}}_2$ with $\sigma _L(\ell _i) = i$. To define $\sigma _R$ and $\sigma _T$, fix an arbitrary $t_0 \in T$. Let $t_1,\dots ,t_{\vert {T} \vert }$ be the elements in *T* with $t_{\vert {T} \vert }=t_0$ such that, for all $j\in {\mathbb {Z}}_{\vert {T} \vert }$, there is some $r_j\in R$ with $\lambda _f(r_j) = (t_j, t_{j+1})$.^6^ Note that these $r_i$ exist since $G_f$ is a cycle. Using this notation, we define $\sigma _T :{\mathbb {Z}}_{\vert {T} \vert } \rightarrow T$ with $\sigma _T(j) = t_j$, for all $j \in {\mathbb {Z}}_{\vert {T} \vert }$. For the elements in *R* we define $\sigma _R:R \rightarrow {\mathbb {Z}}_{\vert {R} \vert }$ with $\sigma _R(r) = j$ whenever $\lambda _f(r)=(t_j, t_{j+1})$ for some *j*. It is easy to check that *f* can be seen as addition modulo $\vert {T} \vert$. Indeed, let $i \in \{0,1\}$ and $r \in R$ with $\lambda _f(r) = (t_j, t_{j+1})$. Then we get
  $$\begin{aligned}{} & {} \sigma _T( \sigma _L(\ell _i) + \sigma _R(r) \bmod \vert {T} \vert ) = \sigma _T(i + j \bmod \vert {T} \vert )\\{} & {} = t_{(i+j \bmod \vert {T} \vert )} = f(\ell _i, r_j) = f(\ell _i, r). \end{aligned}$$
  Thus, *f* is $\vert {T} \vert$-cyclic and $\{ (L, R, \vert {T} \vert ) \}$ is a cyclic partition of *f*.
$$G_f$$ is a path.
Similarly to the previous case, *f* can be represented as addition modulo $\vert {T} \vert$. The proof is essentially identical to the cyclic case and we include it for completeness. Let $\sigma _L :L \rightarrow {\mathbb {Z}}_2$ with $\sigma _L(\ell _i) = i$. Let $t_0,\dots ,t_{\vert {T} \vert -1}$ be the elements of *T* such that, for all $j\in {\mathbb {Z}}_{\vert {T} \vert -1}$, there exist $r_j\in R$ with $\lambda _f(r_j) = (t_j, t_{j+1})$. Since $G_f$ is a path, such $r_j$’s must exist. We let $\sigma _T(j) = t_j$ for every $j \in {\mathbb {Z}}_{\vert {T} \vert }$. We define $\sigma _R:R \rightarrow {\mathbb {Z}}_{\vert {R} \vert }$ with $\sigma _R(r) = j$ whenever $\lambda _f(r)=(t_j, t_{j+1})$ for some *j*. Now, we verify that *f* can be interpreted as addition modulo $\vert {T} \vert$. Consider $i \in \{0,1\}$ and $r \in R$ with $\lambda _f(r) = (t_j, t_{j+1})$ for some $j \in {\mathbb {Z}}_{\vert {T} \vert -1}$. Observe that $j < \vert {T} \vert -1$, hence $t_{j+1 \bmod \vert {T} \vert } = t_{j+1}$. Therefore, we get
  $$\begin{aligned}{} & {} \sigma _T( \sigma _L(\ell _i) + \sigma _R(r) \bmod \vert {T} \vert ) = \sigma _T(i + j \bmod \vert {T} \vert )\\{} & {} = t_{(i+j \bmod \vert {T} \vert )} = f(\ell _i, r_j) = f(\ell _i, r). \end{aligned}$$
  Hence, *f* is $\vert {T} \vert$-cyclic with cyclic partition $\{(L,R,\vert {T} \vert )\}$.

$$\square $$

In the next step, we decompose arbitrary graphs into nice subgraphs. To present our decomposition we need to introduce the following notation related to the degree of vertices.

#### Definition 4.7

(Sources, Sinks and Middle Vertices) Let $G=(V,E)$ be a directed graph. We denote by $\textrm{indeg}(v)$ the *in-degree* of *v*, i.e., the number of edges terminating at *v*, and by $\textrm{outdeg}(v)$ the *out-degree* of *v*, i.e., the number of edges starting at *v*.

We partition *V* into the three sets $V_{\textsf {src} }(G)$, $V_{\textsf {mid} }(G)$, and $V_{\textsf {snk} }(G)$ defined as follows:

- Set $V_{\textsf {src} }(G)$ contains all *source* vertices of *G*, that is, vertices with no incoming edges (i.e., $\textrm{indeg}(v)=0$). This includes all isolated vertices.
- Set $V_{\textsf {mid} }(G)$ contains all *middle* vertices of *G*, that is vertices with incoming and outgoing edges (i.e., $\textrm{indeg}(v),\textrm{outdeg}(v)\ge 1$).
- Set $V_{\textsf {snk} }(G)$ contains the (remaining) *sink* vertices of *G*, that is, vertices with incoming but no outgoing edges (i.e., $\textrm{indeg}(v)\ge 1$ and $\textrm{outdeg}(v)=0$).

We additionally introduce the notion of *deficiency* which we use in the following proofs.

#### Definition 4.8

(Deficiency) Let $G=(V,E)$ be a directed graph. For all $v\in V$, we denote by $\textrm{defi}(v) {:}{=}\max \{ \textrm{outdeg}(v) - \textrm{indeg}(v), 0 \}$ the *deficiency* of *v*.

We define $\textrm{Defi}(G) {:}{=}\sum _{v \in V} \textrm{defi}(v)$ as the *total deficiency* of the graph *G*.

We omit the graph *G* from the notation if it is clear from the context.

We use the deficiency to decompose the acyclic graphs into paths.

#### Lemma 4.9

Every directed graph *G* can be decomposed into $\textrm{Defi}(G)$ paths and an arbitrary number of cycles.

#### Proof

We construct the decomposition ${\mathcal {F}}$ of *G* as follows. In the first phase, we exhaustively find a directed cycle *C* in *G*. We add cycle *C* to the decomposition ${\mathcal {F}}$ and remove the edges of *C* from *G*. We continue the above procedure until graph *G* becomes acyclic. Next, in the second phase we exhaustively find a directed maximum length path *P* (note that *P* may be a single edge). We add *P* to the decomposition ${\mathcal {F}}$ and remove the edges of *P* from *G*. We repeat the second phase until the graph *G* becomes edgeless.

This concludes the construction of decomposition ${\mathcal {F}}$. For correctness observe that the above procedure always terminates because in each step we decrease the number of edges of *G*. Moreover, at the end of the above procedure ${\mathcal {F}}$ is a decomposition of *G* that consists only of paths and cycles.

We are left to show that the number of paths in ${\mathcal {F}}$ is exactly $\textrm{Defi}(G)$. Note that deleting a cycle in *G* does not change the value of $\textrm{Defi}(G)$, hence the first phase of the procedure does not influence $\textrm{Defi}(G)$ and we can assume that *G* is acyclic.

Next, we show that deleting a maximum length path from an acyclic graph decrements its deficiency by exactly 1. This then conclude the proof, because in the second phase of the procedure the deficiency of *G* decreases from $\textrm{Defi}(G)$ down to 0, which means that exactly $\textrm{Defi}(G)$ maximum length paths were added to ${\mathcal {F}}$.

Let *P* be a maximum length, directed path in the acyclic graph *G*. Let $s,t \in V(G)$ be the starting and terminating vertices of path *P*. Path *P* must start at a vertex with a positive deficiency, because otherwise *P* could have been extended at the start which would contradict the fact that *P* is of maximum length. Similarly, since *P* is of maximum length it must terminate in a sink vertex. Hence $\textrm{defi}(s) > 0$ and $\textrm{defi}(t) = 0$. Moreover, every vertex $v \in P {\setminus } \{s,t\}$ has exactly one incoming and one outgoing edge in *P*. Therefore, in the graph $G \setminus P$ the contribution to the total deficiency decreased only in the vertex *s* and only by 1. This means that $\textrm{Defi}(G) = \textrm{Defi}(G {\setminus } P) + 1$ which concludes the proof. $\square$

Now we combine Lemmas 4.6 and 4.9 to show Lemma 4.10.

#### Lemma 4.10

Let $f:L \times R \rightarrow T$ be a function with $\vert {L} \vert =2$ and let $G_f$ be the representation graph of *f*. Then, there exists a cyclic partition ${\mathcal {P}}$ for *f* with $\textrm{cost}({\mathcal {P}}) \le \vert {E(G_f)} \vert + \textrm{Defi}(G_f)$.

#### Proof

First, use Lemma 4.9 to decompose the graph into cycles and $\textrm{Defi}(G_f)$ paths. Then, for each of these paths and cycles, use Lemma 4.6 to obtain the cyclic minor. By Observation 4.5, these minors form a cyclic partition for the function represented by $G_f$. Let ${\mathcal {P}}$ be the resulting cyclic partition.

It remains to analyze the cost of the cyclic partition ${\mathcal {P}}$. By construction, each cyclic minor in ${\mathcal {P}}$ corresponds to a path or a cycle (possibly of length 1). By Lemma 4.6 the cost of a path or a cycle is the number of vertices it contains. Thus, for a path, the cost is equal to the number of edges plus one, and for a cycle the cost is equal to the number of edges. Hence, the cost of ${\mathcal {P}}$ is bounded by the number of edges of $G_f$ plus the number of paths in the decomposition. The latter is precisely $\textrm{Defi}(G_f)$ by Lemma 4.9. $\square$

**Cyclic Partitions Using a Direct Construction** In the following, we use a different method to construct a cyclic partition of the function *f*. Instead of decomposing the graph into nice subgraphs, we directly construct a partition and bound its cost.

#### Lemma 4.11

Let $f:L \times R \rightarrow T$ be a function with $\vert {L} \vert =2$ and let $G_f$ be the representation graph of *f*. Then, there is a cyclic partition ${\mathcal {P}}$ of *f* with $\textrm{cost}({\mathcal {P}}) \le \vert {V(G_f)} \vert + \vert {V_{\textsf {mid} }(G_f)} \vert$.

#### Proof

For each $\ell \in L$, we use a single cyclic minor. Let $L=\{\ell _0, \ell _1\}$. For $i\in \{0,1\}$ define $T_i = \{f(\ell _i,r) \mid r\in R\}$ and $k_i =\vert {T_i} \vert$. Then, ${\mathcal {P}}{:}{=}\{ (\ell _i, R, k_i) \mid i\in \{0,1\}\}$ is the cyclic partition of *f*.

To see that $(\{\ell _i\}, R, k_i)$ is a cyclic minor for $i\in \{0,1\}$, assume w.l.o.g. that $T_i = \{0,1,\ldots , k_i-1\}$ and define $\sigma _L(\ell _i)=0$, $\sigma _R(r) = f(\ell _i,r)$, and $\sigma _T(t) =t$. Thus, ${\mathcal {P}}$ is a cyclic partition of *f* of cost $k_0+k_1 = |T_0|+|T_1|$.

Observe that $\vert {T_0} \vert = \vert {V_{\textsf {src} }(G_f)} \vert + \vert {V_{\textsf {mid} }(G_f)} \vert$ as every $t\in T_0$ has an outgoing edge in $G_f$, and $\vert {T_1} \vert = \vert {V_{\textsf {snk} }(G_f)} \vert + \vert {V_{\textsf {mid} }(G_f)} \vert$ as every $t\in T_1$ has an incoming edge in $G_f$. Hence,

$$\begin{aligned} \textrm{cost}({\mathcal {P}})&= \vert {T_0} \vert +\vert {T_1} \vert \\&= \vert {V_{\textsf {src} }(G_f)} \vert +\vert {V_{\textsf {mid} }(G_f)} \vert + \vert {V_{\textsf {snk} }(G_f)} \vert + \vert {V_{\textsf {mid} }(G_f)} \vert \\&= |V(G_f)| +\vert {V_{\textsf {mid} }(G_f)} \vert \end{aligned}$$

which finishes the proof. $\square$

**Bounding the Cost of Cyclic Partitions** Now, we combine the results from Lemmas 4.10 and 4.11,. We first show how the number of edges relates to the total deficiency of a graph and the number of middle vertices.

#### Lemma 4.12

For every directed graph *G* it holds that $\vert {V_{\textsf {mid} }(G)} \vert + \textrm{Defi}(G) \le \vert {E(G)} \vert$.

#### Proof

Let *m* be the number of edges of *G* and let $e_1,\ldots ,e_m \in E(G)$ be some arbitrarily fixed order of its edges. For every $i \in \{0,\ldots ,m\}$ let $G_i$ be the graph with vertices *V*(*G*) and edges $E(G_i) = \{e_1,\ldots ,e_i\}$. Hence $G_0$ is an independent set of *V*(*G*) and $G_m = G$.

For every $i \in \{0,\ldots ,m\}$ let $\textrm{LHS}(G_i) {:}{=}\vert {V_{\textsf {mid} }(G_i)} \vert + \textrm{Defi}(G_i)$ be the quantity we need to bound. We show that

4.1 $$\begin{aligned} \textrm{LHS}(G_i) - \textrm{LHS}(G_{i-1}) \le 1 \text { for every } i \in [m] \end{aligned}$$

which then concludes the proof because

$$\begin{aligned} \vert {V_{\textsf {mid} }(G)} \vert + \textrm{Defi}(G) = \textrm{LHS}(G_m) = \sum _{i=1}^m \left( \textrm{LHS}(G_i) - \textrm{LHS}(G_{i-1})\right) \le m = \vert {E(G)} \vert . \end{aligned}$$

From now, we focus on the proof of Eq. 4.1. For every $v \in V(G)$ and $i \in \{0,\ldots ,m\}$, let $\textrm{defi}_i(v)$ be the deficiency of vertex *v* in graph $G_i$. Next, for every $v \in V(G)$ and $i \in [m]$, we define

$$\begin{aligned} \Delta _i(v) {:}{=}\textrm{defi}_i(v) - \textrm{defi}_{i-1}(v) + \llbracket {v \in V_{\textsf {mid} }(G_i) \setminus V_{\textsf {mid} }(G_{i-1})}\rrbracket \end{aligned}$$

Consider a step $i \in [m]$. Let $e_i = (s,t)$ be an *i*th edge that starts at a vertex *s* and terminates at a vertex *t*. It holds that

$$\begin{aligned} \vert {V_{\textsf {mid} }(G_i)} \vert + \textrm{Defi}(G_i) = \vert {V_{\textsf {mid} }(G_{i-1})} \vert + \textrm{Defi}(G_{i-1}) + \Delta _i(s) + \Delta _i(t). \end{aligned}$$

Therefore $\textrm{LHS}(G_i) - \textrm{LHS}(G_{i-1}) = \Delta _i(s) + \Delta _i(t)$ and to establish Eq. 4.1 it is enough to show that $\Delta _i(s) \le 1$ and $\Delta _i(t) \le 0$.

#### Claim 4.13

It holds that $\Delta _i(s) \le 1$.

#### Proof

We consider two cases depending on whether *u* became a middle vertex. If it happened that $s \in V_{\textsf {mid} }(G_i) {\setminus } V_{\textsf {mid} }(G_{i-1})$, then $s \in V_{\textsf {snk} }(G_{i-1})$ which means that *s* has more incoming than outgoing edges in $G_{i-1}$. Hence $\textrm{defi}_{i-1}(s) = \textrm{defi}_i(s) = 0$ and we conclude that $\Delta _i(s)=1$.

Otherwise $s \notin V_{\textsf {mid} }(G_i)\setminus V_{\textsf {mid} }(G_{i-1})$. Because the edge $e_i$ starts at *s*, the deficiency of *s* can increase by at most 1. Hence, by $(\textrm{defi}_i(s) - \textrm{defi}_{i-1}(s)) \le 1$ we conclude that $\Delta _i(s) \le 1$. $\square$

Finally, we consider the end vertex *t* of the edge $e_i$.

#### Claim 4.14

It holds that $\Delta _i(t) \le 0$.

#### Proof

We again distinguish two cases depending on whether *t* became a middle vertex. If $t \in V_{\textsf {mid} }(G_i) \setminus V_{\textsf {mid} }(G_{i-1})$, then $t \in V_{\textsf {src} }(G_{i-1})$ and moreover, *t* has no incoming edges and the positive number of outgoing edges in $G_{i-1}$. Therefore $\textrm{defi}_i(t) = \textrm{defi}_{i-1}(t)-1$ which means that $\Delta _i(t) \le 0$.

It remains to analyse the case when $t \notin V_{\textsf {mid} }(G_i) {\setminus } V_{\textsf {mid} }(G_{i-1})$. Since the edge $e_i$ ends at *t*, the deficiency of *t* cannot increase and $\textrm{defi}_i(v) \le \textrm{defi}_{i-1}(v)$. This means that $\Delta _i(t) \le 0$. $\square$

By Claims 4.13 and 4.14, it follows that $\Delta _i(s) + \Delta _i(t) \le 1$. This establishes Eq. 4.1 and concludes the proof. $\square$

Now we are ready to combine Lemmas 4.10 and 4.11, and prove Lemma 4.2.

#### Proof of Lemma 4.2

As before, we denote by $G_f$ the representation graph of *f*. Let *V* and *E* be the set of vertices and edges of graph $G_f$.

Let ${\mathcal {P}}_1$ be the cyclic partition of *f* from Lemma 4.10 with cost at most $\vert {E} \vert +\textrm{Defi}(G_f)$ and let ${\mathcal {P}}_2$ be the cyclic partition of *f* from Lemma 4.11 with cost at most $\vert {V} \vert + \vert {V_{\textsf {mid} }(G_f)} \vert$.

We define ${\mathcal {P}}$ as the minimum cost partition among ${\mathcal {P}}_1$ and ${\mathcal {P}}_2$. This implies that

$$\begin{aligned} \textrm{cost}({\mathcal {P}})&\le \min \{ \textrm{cost}({\mathcal {P}}_1),\textrm{cost}({\mathcal {P}}_2) \} \le \frac{\textrm{cost}({\mathcal {P}}_1) + \textrm{cost}({\mathcal {P}}_2)}{2} \\&\le \frac{\vert {E} \vert + \vert {V} \vert + \vert {V_{\textsf {mid} }(G_f)} \vert + \textrm{Defi}(G_f)}{2}. \end{aligned}$$

Next, we use the inequality $\vert {V_{\textsf {mid} }(G_f)} \vert + \textrm{Defi}(G_f)\le \vert {E} \vert$ from Lemma 4.12, and get

$$\begin{aligned} \textrm{cost}({\mathcal {P}})&\le \vert {E} \vert + \frac{\vert {V} \vert }{2}. \end{aligned}$$

Since $\vert {E} \vert \le \vert {R} \vert$ and $\vert {V} \vert =\vert {T} \vert$ this concludes the proof. $\square$

### General Case: Proof of Lemma 4.1

Now we have everything ready to prove the main result of this section.

#### Proof of Lemma 4.1

We first handle the case when $\vert {L} \vert$ is even. We partition *L* into $\lambda =\vert {L} \vert /2$ sets $L_1,\dots ,L_\lambda$ consisting of exactly two elements. We use Lemma 4.2 to find a cyclic partition ${\mathcal {P}}_i$ for each $f_i:L_i \times R \rightarrow T$. By definition of the cyclic partition, ${\mathcal {P}}= \bigcup _{i\in [\lambda ]} {\mathcal {P}}_i$ is a cyclic partition for *f*, hence it remains to analyze the cost of ${\mathcal {P}}$.

Observe that for each $G_i$ we have that $\vert {V_i} \vert \le \vert {T} \vert$ and $\vert {E_i} \vert \le \vert {R} \vert$. By the definition of the cost of the cyclic partition, we immediately get that

$$\begin{aligned} \textrm{cost}({\mathcal {P}}) \le \sum _{i=1}^\lambda \textrm{cost}({\mathcal {P}}_i) \le \lambda \cdot \left( \vert {R} \vert + \frac{\vert {T} \vert }{2}\right) . \end{aligned}$$

If $\vert {L} \vert$ is odd, then we remove one element $\ell$ from *L* and let $L_0=\{\ell \}$. There is a trivial cyclic partition ${\mathcal {P}}_0$ for $f_0:L_0\times R \rightarrow T$ of cost at most $\vert {R} \vert$. Then we use the above procedure to find a cyclic partition ${\mathcal {P}}'$ for the restriction of *f* to $L{\setminus }\{\ell \}$ and *R*. Hence, setting ${\mathcal {P}}= {\mathcal {P}}_0 \cup {\mathcal {P}}'$ gives a cyclic partition for *f* with cost

$$\begin{aligned} \textrm{cost}({\mathcal {P}}) \le \textrm{cost}({\mathcal {P}}_0) + \textrm{cost}({\mathcal {P}}') \le \vert {R} \vert + {\left\lfloor \frac{\vert {L} \vert }{2} \right\rfloor } \left( \vert {R} \vert + \frac{\vert {T} \vert }{2} \right) . \end{aligned}$$

$$\square $$

#### Remark 4.15

If $\vert {L} \vert$ and $\vert {R} \vert$ are both even, one can easily achieve a cost of

$$\begin{aligned} \min \left( \frac{L}{2} \cdot \left( \vert {R} \vert + \frac{\vert {T} \vert }{2} \right) , \frac{R}{2} \cdot \left( \vert {L} \vert + \frac{\vert {T} \vert }{2} \right) \right) = \frac{\vert {L} \vert \cdot \vert {R} \vert }{2} + \frac{\vert {T} \vert }{4} \cdot \min (\vert {L} \vert ,\vert {R} \vert ) \end{aligned}$$

by swapping the role of *L* and *R* and considering the function $f':R \times L \rightarrow T$ with $f'(r,\ell )=f(\ell ,r)$ for all $\ell \in L$ and $r \in R$.

### Tight Example: Lower Bound on Lemma 4.2

To complement the previous results, we show that Lemma 4.2 is tight. That is, there is a function $f:L\times R\rightarrow T$ with $\vert {L} \vert =2$ such that no cyclic partition ${\mathcal {P}}$ of *f* has smaller cost, i.e., $\textrm{cost}({\mathcal {P}}) < \vert {R} \vert + \vert {T} \vert /2$. In particular, this demonstrates that to improve the constant $c {:}{=}3/4$ in Theorem 1.1 new ideas are needed.

**Fig. 3:**
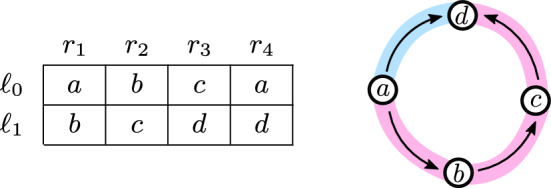
The definition of the function *f* used by Lemma 4.16, which shows that the bound from Lemma 4.2 is tight. The representation graph of *f* is depicted on the right. We highlight the cyclic partition returned by Lemma 4.16. The red path contains 4 vertices and the blue path contains 2 vertices. Hence, the cost of that cyclic partition is 6. Lemma 4.16 shows that this is the best possible (Color figure online)

#### Lemma 4.16

There exist sets *L*, *R*, and *T* with $\vert {L} \vert =2$ and a function $f:L\times R\rightarrow T$ such that, every cyclic partition ${\mathcal {P}}$ of *f* has $\textrm{cost}({\mathcal {P}}) \ge \vert {R} \vert + \vert {T} \vert /2$.

#### Proof

Define $L =\{\ell _0,\ell _1\}$, $R=\{r_1,r_2,r_3,r_4\}$, and $T=\{a,b,c,d\}$. Let *f* be the function as defined in Fig. 3. Note that we need to show that every cyclic partition of *f* has cost at least 6.

Let ${\mathcal {P}}$ be a cyclic partition of *f*. We first claim that the cyclic partition ${\mathcal {P}}$ of *f* contains a single cyclic minor, i.e., ${\mathcal {P}}=\{(L,R,k)\}$ for some integer *k*. For contradictions sake, we analyse every other remaining structure of ${\mathcal {P}}$ and argue that in each case $\textrm{cost}({\mathcal {P}})\ge 6=\vert {R} \vert +\vert {T} \vert /2$.

- Every cyclic minor in ${\mathcal {P}}$ is of the form $(\{\ell _i\}, B, k)$ (i.e., uses only values from a single row). Then, $\textrm{cost}({\mathcal {P}})\ge 6$ as each row has 3 distinct values.
- There is a cyclic minor $(\{\ell _0,\ell _1\}, \{r_j\}, k)$ in ${\mathcal {P}}$. Since each column contains two distinct elements, It must hold that $k\ge 2$. Furthermore, the cyclic minors which cover the remainder of the graph must have a total cost of 4 (or more) as all values in *T* appear in the remainder of the graph. Hence $\textrm{cost}({\mathcal {P}})\ge 6$.
- There is a cyclic minor $(\{\ell _0, \ell _1\}, \{r_j,r_{j'}\},k)$ in ${\mathcal {P}}$. Since each pair of two columns contains (at least) three values, it must hold that $k\ge 3$. There are at least 3 distinct values in the remainder of the graph, hence, the cost of the remaining minors in ${\mathcal {P}}$ is at least 3. Thus $\textrm{cost}({\mathcal {P}})\ge 6$.
- There is a cyclic minor $(\{\ell _0, \ell _1\}, R\setminus \{r_j\},k)$ in ${\mathcal {P}}$. It holds that $k\ge 4$ as every three columns include all values in *T*. In each case, there are two different values in the remaining column. Hence, the cost of the remaining minors is at least 2. Therefore $\textrm{cost}({\mathcal {P}})\ge 6$.

With this, we know that ${\mathcal {P}}$ contains only the single cyclic minor (*L*, *R*, *k*). Let $\sigma _L, \sigma _R$ and $\sigma _T$ be the relabelling functions of (*L*, *R*, *k*). From the definition of the relabeling functions, we get that $F{:}{=}\{ \sigma _L(\ell _i) + \sigma _R(r_j) \mod k \mid i \in \{0,1\} \text { and } j\in \{1,2,3\} \}$ contains at least four elements.

We claim that $(\sigma _L(\ell _0)+\sigma _R(r_4) \mod k) \notin F$. For the sake of contradiction assume otherwise. Then, by the definition of $\sigma _T$, it must hold that $\sigma _L(\ell _0)+\sigma _R(r_1) \equiv _k \sigma _L(\ell _0)+\sigma _R(r_4)$. As this implies $\sigma _R(r_1)=\sigma _R(r_4)$, we get

$$\begin{aligned} b = f(\ell _1,r_1 ) =\,&\sigma _T( \sigma _L(\ell _1) + \sigma _R(r_1) \mod k ) \\ =\,&\sigma _T( \sigma _L(\ell _1) + \sigma _R(r_4) \mod k ) = f(\ell _1,r_4) = d, \end{aligned}$$

which is a contradiction.

Similarly, we get that $(\sigma _L(\ell _1)+\sigma _R(r_4) \mod k) \notin F$. Again assuming otherwise, we have that $\sigma _R(r_3)=\sigma _R(r_4)$ which then implies

$$\begin{aligned} c = f(\ell _0, r_3) =\,&\sigma _T( \sigma _L(\ell _0) + \sigma _R(r_3) \mod k ) \\ =\,&\sigma _T( \sigma _L(\ell _0) + \sigma _R(r_4) \mod k ) = f(\ell _0, r_4) = a, \end{aligned}$$

which is a contradiction.

Since, $F \cup \{ \sigma _L(\ell _0)+\sigma _R(r_4) \mod k, \sigma _L(\ell _1)+\sigma _R(r_4) \mod k \} \subseteq {{\mathbb {Z}}}_k$, contains at least six distinct elements, we get $k \ge 6$ and therefore, $\textrm{cost}({\mathcal {P}}) \ge 6$. $\square$

## Querying a Generalized Convolution

In this section, we prove Theorem 1.4. The main idea is to represent the $f$-Query problem as a matrix multiplication problem, inspired by a graph interpretation of $f$-Query.

Let $D$ be an arbitrary set and $f:D\times D\rightarrow D$. We assume *D* and *f* are fixed throughout this section. Let $g,h:D^n\rightarrow {\{-M,\ldots , M\}}$ and $\textbf{v}\in D^n$ be a $f$-Query instance. We use $\textbf{a}\Vert \textbf{b}$ to denote the concatenation of $\textbf{a}\in D^{m}$ and $\textbf{b}\in D^{k}$. That is $(\textbf{a}_1,\ldots , \textbf{a}_{m})\Vert (\textbf{b}_1,\ldots ,\textbf{b}_{k}) = (\textbf{a}_1,\ldots , \textbf{a}_{m}, \textbf{b}_1,\ldots , \textbf{b}_{k})$. If we assume that *n* is even, then, for a vector $\textbf{v}\in D^n$, let $\textbf{v}^{(\textrm{high})},\textbf{v}^{{(\textrm{low})}}\in D^{n/2}$ be the unique vectors such that $\textbf{v}^{{(\textrm{high})}} \Vert \textbf{v}^{{(\textrm{low})}} = \textbf{v}$. Indeed, to achieve this assumption let *n* be odd, fix an arbitrary $d \in D$, and define ${\widetilde{g}}, {\widetilde{h}} :D^{n+1}\rightarrow {\{-M,\ldots , M\}}$ as ${\widetilde{g}}(\textbf{u}_1,\ldots \textbf{u}_{n+1}) = \llbracket {\textbf{u}_{n+1} = d}\rrbracket \cdot g(\textbf{u}_1,\ldots \textbf{u}_{n})$ and ${\widetilde{h}}(\textbf{u}_1,\ldots \textbf{u}_{n+1}) = \llbracket {\textbf{u}_{n+1} = d}\rrbracket \cdot h(\textbf{u}_1,\ldots \textbf{u}_{n})$ for all $\textbf{u}\in D^{n+1}$. It can be easily verified that $(g\mathbin {\circledast _{f}}h)(\textbf{v}) = ({\widetilde{g}} \mathbin {\circledast _{f}}{\widetilde{h}})(\textbf{v}\Vert (f(d,d)))$. Thus, we can solve the $f$-Query instance ${\widetilde{g}}$, ${\widetilde{h}}$ and $\textbf{v}\Vert (f(d,d))$ and obtain the correct result.

We first provide the intuition behind the algorithm and then formally present the algorithm and show correctness.

**Intuition** We define a directed multigraph *G* where the vertices are partitioned into four layers $\text {L}^{{(\textrm{high})}}$, $\text {L}^{{(\textrm{low})}}$, $\text {R}^{{(\textrm{low})}}$, and $\text {R}^{{(\textrm{high})}}$. Each of these sets consists of $|D|^{n/2}$ vertices representing every vector in $D^{n/2}$. For ease of notation, we use the vectors to denote the associated vertices; furthermore, the intuition assumes *g* and *h* are non-negative. The multigraph *G* contains the following edges:

- $g(\textbf{w}\Vert \textbf{x})$ parallel edges from $\textbf{w}\in D^{n/2}$ in $\text {L}^{{(\textrm{high})}}$ to $\textbf{x}\in D^{n/2}$ in $\text {L}^{{(\textrm{low})}}$.
- One edge from $\textbf{x}\in D^{n/2}$ in $\text {L}^{{(\textrm{low})}}$ to $\textbf{y}\in D^{n/2}$ in $\text {R}^{{(\textrm{low})}}$ if and only if $\textbf{x}\oplus _f \textbf{y}=v^{(\textrm{low})}$.
- $h(\textbf{z}\Vert \textbf{y})$ parallel edges from $\textbf{y}\in D^{n/2}$ in $\text {R}^{{(\textrm{low})}}$ to $\textbf{z}\in D^{n/2}$ in $\text {R}^{{(\textrm{high})}}$.
- One edge from $\textbf{z}\in D^{n/2}$ in $\text {R}^{{(\textrm{high})}}$ to $\textbf{w}\in D^{n/2}$ in $\text {L}^{{(\textrm{high})}}$ if and only if $\textbf{w}\oplus _f\textbf{z}=v^{(\textrm{high})}$.

**Fig. 4:**
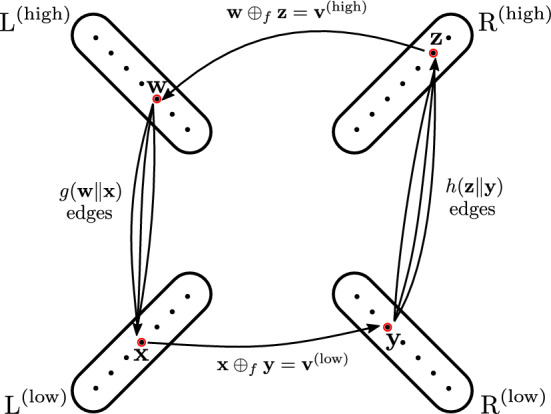
Construction of the directed multigraph *G*. Each vertex in a layer corresponds to the vector in $D^{n/2}$. We highlighted 4 vectors $\textbf{w},\textbf{x},\textbf{y},\textbf{z}\in D^{n/2}$ each in a different layer. Note that the number of 4 cycles that go through all four $\textbf{w},\textbf{x},\textbf{y},\textbf{z}$ is equal to $g(\textbf{w}\Vert \textbf{x}) \cdot h(\textbf{z}\Vert \textbf{y})$. The total number of directed 4-cycles in this graph corresponds to the value $(g\mathbin {\circledast _{f}}h)(\textbf{v})$ and $\text {tr}(W\cdot X \cdot Y\cdot Z)$

In the formal proof, we denote the adjacency matrix between $\text {L}^{{(\textrm{high})}}$ and $\text {L}^{{(\textrm{low})}}$ by *W*, between $\text {L}^{{(\textrm{low})}}$ and $\text {R}^{{(\textrm{low})}}$ by *X*, between $\text {R}^{{(\textrm{low})}}$ and $\text {R}^{{(\textrm{high})}}$ by *Y*, and between $\text {R}^{{(\textrm{high})}}$ and $\text {L}^{{(\textrm{high})}}$ by *Z*. See Fig. 4 for an example of this construction.

Let $\textbf{w}, \textbf{x}, \textbf{y},\textbf{z}\in D^{n/2}$ be vertices in $\text {L}^{{(\textrm{high})}}$, $\text {L}^{{(\textrm{low})}}$, $\text {R}^{{(\textrm{low})}}$, and $\text {R}^{{(\textrm{high})}}$. It can be observed that if $(\textbf{w}\Vert \textbf{x}) \oplus _f (\textbf{y}\Vert \textbf{z}) \ne \textbf{v}$, then *G* does not contain any cycle of the form $\textbf{w}\rightarrow \textbf{x}\rightarrow \textbf{y}\rightarrow \textbf{z}\rightarrow \textbf{w}$ as one of the edges $(\textbf{x}, \textbf{y})$ or $(\textbf{z}, \textbf{w})$ is not present in the graph. Conversely, if $(\textbf{w}\Vert \textbf{x})\oplus _f (\textbf{y}\Vert \textbf{z})= \textbf{v}$, then one can verify that there are $g(\textbf{w}\Vert \textbf{x})\cdot h(\textbf{z}\Vert \textbf{y})$ cycles of the form $\textbf{w}\rightarrow \textbf{x}\rightarrow \textbf{y}\rightarrow \textbf{z}\rightarrow \textbf{w}$. We therefore expect that $(g \mathbin {\circledast _{f}}h)(\textbf{v})$ is the number of cycles in *G* that start at some $\textbf{w}\in D^{n/2}$ in $\text {L}^{{(\textrm{high})}}$, have length four, and end at the same vertex $\textbf{w}$ in $\text {L}^{{(\textrm{high})}}$ again.

**Formal Proof** We use the notation $\textsf{Mat}_{\mathbb {Z}}(D^{n/2}\times D^{n/2})$ to refer to a $|D|^{n/2} \times |D|^{n/2}$ matrix of integers where we use the values in $D^{n/2}$ as indices. The *transition matrices* of *g*, *h* and $\textbf{v}$ are the matrices $W,X,Y,Z\in \textsf{Mat}_{\mathbb {Z}}(D^{n/2}\times D^{n/2})$ defined by

$$\begin{aligned} W_{\textbf{w},\textbf{x}}&{:}{=}g(\textbf{w}\Vert \textbf{x}){} & {} \forall \textbf{w},\textbf{x}\in D^{n/2} \\ X_{\textbf{x},\textbf{y}}&{:}{=}\llbracket { \textbf{x}\oplus _f \textbf{y}=\textbf{v}^{(\textrm{low})}}\rrbracket{} & {} \forall \textbf{x},\textbf{y}\in D^{n/2} \\ Y_{\textbf{y},\textbf{z}}&{:}{=}h(\textbf{z}\Vert \textbf{y}){} & {} \forall \textbf{y},\textbf{z}\in D^{n/2} \\ Z_{\textbf{z},\textbf{w}}&{:}{=}\llbracket { \textbf{w}\oplus _f\textbf{z}=\textbf{v}^{(\textrm{high})}}\rrbracket{} & {} \forall \textbf{z},\textbf{w}\in D^{n/2} \end{aligned}$$

Recall that the *trace* $\text {tr}(A)$ of a matrix $A \in \textsf{Mat}_{\mathbb {Z}}(m \times m)$ is defined as $\text {tr}(A) {:}{=}\sum _{i=1}^m A_{i,i}$. The next lemma formalizes the correctness of this construction.

### Lemma 5.1

Let $n\in {\mathbb {N}}$ be an even number, $g,h :D^n\rightarrow {\mathbb {Z}}$ and $\textbf{v}\in D^n$. Also, let $W,X,Y,Z\in \textsf{Mat}_{\mathbb {Z}}(D^{n/2}\times D^{n/2})$ be the transition matrices of *g*, *h* and $\textbf{v}$. Then,

$$\begin{aligned} (g\mathbin {\circledast _{f}}h) (\textbf{v}) = \text {tr}(W\cdot X \cdot Y\cdot Z). \end{aligned}$$

### Proof

For any $\textbf{w}, \textbf{y}\in D^{n/2}$ it holds that,

5.1 $$\begin{aligned} (W\cdot X)_{\textbf{w},\textbf{y}} = \sum _{\textbf{x}\in D^{n/2}} W_{\textbf{w},\textbf{x}}\cdot X_{\textbf{x}, \textbf{y}} = \sum _{ \textbf{x}\in D^{n/2}} \llbracket {\textbf{x}\oplus _f \textbf{y}= \textbf{v}^{(\textrm{low})}}\rrbracket \cdot g(\textbf{w}\Vert \textbf{x}). \end{aligned}$$

Similarly, for any $\textbf{y},\textbf{w}\in D^{n/2}$ it holds that,

5.2 $$\begin{aligned} (Y\cdot Z)_{\textbf{y},\textbf{w}} = \sum _{\textbf{z}\in D^{n/2}} Y_{\textbf{y},\textbf{z}}\cdot Z_{\textbf{z}, \textbf{w}} = \sum _{ \textbf{z}\in D^{n/2}} \llbracket {\textbf{w}\oplus _f\textbf{z}= \textbf{v}^{(\textrm{high})}}\rrbracket \cdot h(\textbf{z}\Vert \textbf{y}). \end{aligned}$$

Therefore, for any $\textbf{w}\in D^{n/2}$,

$$\begin{aligned}&(W\cdot X\cdot Y\cdot Z)_{\textbf{w},\textbf{w}} = \sum _{\textbf{y}\in D^{n/2}} (W\cdot X)_{\textbf{w},\textbf{y}}\cdot (Y\cdot Z)_{\textbf{y}, \textbf{w}} \\&\quad = \sum _{\textbf{y}\in D^{n/2}} \left( \sum _{ \textbf{x}\in D^{n/2}}\llbracket { \textbf{x}\oplus _f \textbf{y}= \textbf{v}^{(\textrm{low})}}\rrbracket \cdot g(\textbf{w}\Vert \textbf{x}) \right) \left( \sum _{ \textbf{z}\in D^{n/2}} \llbracket { \textbf{w}\oplus _f\textbf{z}= \textbf{v}^{(\textrm{high})}}\rrbracket \cdot h(\textbf{z}\Vert \textbf{y}) \right) \\&\quad =\sum _{\textbf{x},\textbf{y},\textbf{z}\in D^{n/2}} \llbracket { \textbf{x}\oplus _f\textbf{y}=\textbf{v}^{(\textrm{low})}}\rrbracket \cdot \llbracket {\textbf{w}\oplus _f\textbf{z}=\textbf{v}^{(\textrm{high})}~}\rrbracket \cdot g(\textbf{w}\Vert \textbf{x}) \cdot h(\textbf{z}\Vert \textbf{y}) \\&\quad =\sum _{\textbf{x},\textbf{y},\textbf{z}\in D^{n/2}} \llbracket { (\textbf{w}\Vert \textbf{x})\oplus _f(\textbf{z}\Vert \textbf{y})=\textbf{v}^{(\textrm{high})}\Vert \textbf{v}^{(\textrm{low})}}\rrbracket \cdot g(\textbf{w}\Vert \textbf{x}) \cdot h(\textbf{z}\Vert \textbf{y}), \end{aligned}$$

where the second equality follows by (5.1) and (5.2). Thus,

$$\begin{aligned} \text {tr}(W\cdot X~\cdot ~ Y\cdot Z )&= \sum _{\textbf{w}\in D^{n/2}} (W\cdot X\cdot Y\cdot Z)_{\textbf{w},\textbf{w}} \\&= \sum _{\textbf{w}\in D^{n/2} }\sum _{~\textbf{x},\textbf{y},\textbf{z}\in D^{n/2}} \llbracket { (\textbf{w}\Vert \textbf{x})\oplus _f (\textbf{z}\Vert \textbf{y})=\textbf{v}}\rrbracket \cdot g(\textbf{w}\Vert \textbf{x}) \cdot h(\textbf{z}\Vert \textbf{y}) \\&= \sum _{\textbf{u}, \textbf{t}\in D^{n}} \llbracket {\textbf{u}\oplus _f \textbf{t}= \textbf{v}}\rrbracket \cdot g(\textbf{u})\cdot h(\textbf{t}) \\&= (g\mathbin {\circledast _{f}}h) (\textbf{v}). \end{aligned}$$

$$\square $$

Now we have everything ready to give the algorithm for $f$-Query.

### Proof of Theorem 1.4

The algorithm for solving $f$-Query works in two steps:

1. Compute the transition matrices *W*, *X*, *Y*, and *Z* of *g*, *h* and $\textbf{v}$ as described above.
2. Compute and return $\text {tr}(W \cdot X \cdot Y \cdot Z)$.

By Lemma 5.1 this algorithm returns $(g\mathbin {\circledast _{f}}h)(\textbf{v})$. Computing the transition matrices in Step 1. requires $\widetilde{{\mathcal {O}}}(|D|^n \cdot \textrm{polylog}(M))$ time. Observe the maximal absolute values of an entry in the transition matrices is *M*. The computation of $W\cdot X\cdot Y\cdot Z$ in Step 2. requires three matrix multiplications of $|D|^{n/2}\times |D|^{n/2}$ matrices, which can be done in $\widetilde{{\mathcal {O}}}((|D|^{n /2})^\omega \cdot \textrm{polylog}(M))$ time. Thus, the overall running time of the algorithm is $\widetilde{{\mathcal {O}}}(|D|^{\omega \cdot n / 2} \cdot \textrm{polylog}(M))$. $\square$

## Conclusion and Future Work

In this paper, we studied the $f$-Convolution problem and demonstrated that the naive brute-force algorithm can be improved for every $f :D \times D \rightarrow D$. We achieve that by introducing a *cyclic partition* of a function and showing that there always exists a cyclic partition of bounded cost. We give an $\widetilde{{\mathcal {O}}}((c|D|^2)^{n} \cdot \textrm{polylog}(M))$ time algorithm that computes $f$-Convolution for $c {:}{=}3/4$ when $|D|$ is even.

The cyclic partition is a very general tool and potentially it can be used to achieve greater improvements for certain functions *f*. For example, in multiple applications (e.g., [19, 23, 29, 34]) the function *f* has a cyclic partition with a single cyclic minor. Nevertheless, in our proof we only use cyclic minors where one domain is of size is at most 2. We suspect that larger minors have to be considered to obtain better results. Indeed, the lower bound from Lemma 4.16 implies that our technique of considering two *arbitrary* rows together cannot give a faster algorithm than $\widetilde{{\mathcal {O}}}((3/4 \cdot \vert {D} \vert ^2)^n \cdot \textrm{polylog}(M))$ in general. An improved algorithm would have to select these rows very carefully or consider three or more rows at the same time.

We leave several open problems. Our algorithm offers an exponential (in *n*) improvement over a naive algorithm for domains $D$ of constant size. Can we hope for an $\widetilde{{\mathcal {O}}}(|D|^{(2-\epsilon )n} \cdot \textrm{polylog}(M))$ time algorithm for $f$-Convolution for some $\epsilon > 0$? We are not aware of any lower bounds, so in principle even an $\widetilde{{\mathcal {O}}}(|D|^n \cdot \textrm{polylog}(M))$ time algorithm is plausible.

**Fig. 5:**
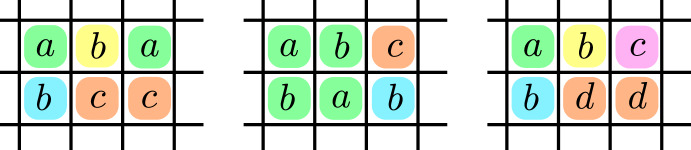
Here are three concrete examples of functions *f* for which we expect that the running times for $f$-Convolution should be $\widetilde{{\mathcal {O}}}(3^n \cdot \textrm{polylog}(M))$, $\widetilde{{\mathcal {O}}}(3^n \cdot \textrm{polylog}(M))$ and $\widetilde{{\mathcal {O}}}(4^n \cdot \textrm{polylog}(M))$. However, the best cyclic partitions for this functions have costs 4, 4 and 5 (the partitions are highlighted appropriately). This implies that the best running time, which may be attained using our techniques are $\widetilde{{\mathcal {O}}}(4^n \cdot \textrm{polylog}(M))$, $\widetilde{{\mathcal {O}}}(4^n \cdot \textrm{polylog}(M))$ and $\widetilde{{\mathcal {O}}}(5^n \cdot \textrm{polylog}(M))$ (Color figure online)

Ideally, we would expect that the $f$-Convolution problem can be solved in $\widetilde{{\mathcal {O}}}((|L|^n+|R|^n+|T|^n) \cdot \textrm{polylog}(M))$ for any function $f :L \times R \rightarrow T$. In Fig. 5 we include three examples of functions that are especially difficult for our methods.

Finally, we gave an $\widetilde{{\mathcal {O}}}(|D|^{\omega \cdot n / 2} \cdot \textrm{polylog}(M))$ time algorithm for $f$-Query problem. For $\omega = 2$ this algorithm runs in almost linear-time, however for the current bound $\omega < 2.372$ our algorithm runs in time $\widetilde{{\mathcal {O}}}(|D|^{1.19n} \cdot \textrm{polylog}(M))$. Can $f$-Query be solved in $\widetilde{{\mathcal {O}}}(|D|^n \cdot \textrm{polylog}(M))$ time without assuming $\omega =2$?

## A Proof of Theorem 2.6

In this section we prove Theorem 2.6. We crucially rely on the following result by van Rooij [33].

### Theorem A.1

([33, Lemma 3]) There is an algorithm which given $k\in {\mathbb {N}}$, $\textbf{r}\in {\mathbb {N}}^k$ a prime *p*, an $\textbf{r}_j$-th primitive root of unity $\omega _j$ for every $j\in [k]$ and two functions $g,h:{\mathbb {Z}}_{\textbf{r}_1} \times \dots \times {\mathbb {Z}}_{\textbf{r}_k} \rightarrow {\mathbb {Z}}$ computes the cyclic convolution of *g* and *h* modulo *p* (that is, return a function $\phi$ such that $\phi (\textbf{q})=(g \odot h)(\textbf{v}) \mod p$ for every $\textbf{v}\in {\mathbb {Z}}_{r_1}\times \dots \times {\mathbb {Z}}_{r_k}$) in ${\mathcal {O}}( R\log (R))$ arithmetic operations where $R=\prod _{j=1}^k \textbf{r}_j$.

Ideally, we would like to use the algorithm from Theorem A.1 with a sufficiently large prime *p* such that the values of $g \mathbin {\odot }h$ could be recovered from the values of $g \mathbin {\odot }h$ modulo *p*. Finding such a prime *p* along with the required roots of unity is, however, a non trivial task which we do not know how to perform deterministically while retaining the running time at $\widetilde{{\mathcal {O}}}\left( R \cdot \textrm{polylog}(M)\right)$. The basic idea behind our approach is to compute $g \odot h$ modulo $p_i$ for a sufficiently large number of distinct small primes $p_i$ using Theorem A.1. If $\prod _i p_i$ is sufficiently large, then the values of $g \odot h$ can be uniquely recovered using the Chinese Remainder Theorem.

### Theorem A.2

(Chinese Remainder Theorem) Let $p_1,\dots ,p_m$ denote a sequence of integers that are pairwise coprime and define $P {:}{=}\prod _{i \in [m]}p_i$. Also let $0 \le a_i < p_i$ for all $i \in [m]$. Then there is a unique number $0 \le s < P$ such that

$$\begin{aligned} s \equiv a_i \mod p_i \end{aligned}$$

for all $i \in [m]$. Moreover, there is an algorithm that, given $p_1,\dots ,p_m$ and $a_1,\dots ,a_m$, computes the number *s* in time ${\mathcal {O}}((\log P)^2)$.

To find the small primes for the application of the Chinese Remainder Theorem, we additionally use density properties of primes in arithmetic progression. Given $q\in {\mathbb {N}}$, we say $p\in {\mathbb {N}}$ is a *q*-*prime* if *p* is a prime number and $p\equiv 1 \mod q$. We use ${\textsf {prime}}_q(i)$ to denote the *i*-th *q*-prime. That is, ${\textsf {prime}}_q(i)$ is a *q*-prime such that the number of *q*-primes smaller than ${\textsf {prime}}_q(i)$ is exactly $i-1$. Also, for any $B,q\in {\mathbb {N}}$, we define

$$\begin{aligned} {\textsf {prime\_bound}}_q(B) {:}{=}\min \left\{ m\in {\mathbb {N}}~\big |~\prod _{i=1}^{m} {\textsf {prime}}_q(i) \ge B \right\} \end{aligned}$$

to be the minimal number *m* such that the product of the first *m* *q*-primes is at least *B*. We use the following upper bound on ${\textsf {prime\_bound}}$.

### Lemma A.3

Let $B, q \in {\mathbb {N}}$ be integers such that $B,q\ge 3$ and $m=\text {{\textsf {prime\_bound}}}_q(B)$. Then $m\le \ln (B)+1$ and $\text {{\textsf {prime}}}_q(m) \le \max \big \{ \exp \left( 8\cdot \sqrt{q} \cdot \ln ^3(q) \right) ,~ \exp (q), ~2q\cdot \ln (B) \big \}$.

In the proof of Lemma A.3 we use a known result for the density of primes in arithmetic progressions taken from [3]. For any $x,q \in {\mathbb {N}}$, define $\theta (x,q)$ to be the sum of $\ln (p)$ for all *q*-primes *p* such that $p\le x$. Formally, we define

$$\begin{aligned} \theta (x,q) {:}{=}\sum _{i=1}^{\infty } \llbracket {{\textsf {prime}}_q(i) \le x }\rrbracket \cdot \ln \left( {\textsf {prime}}_q(i)\right) . \end{aligned}$$

With this definition, we can now state the result about the density of primes in arithmetic progressions.

### Lemma A.4

([3, Corollary 1.8]) Let *q* and *x* be integers with $q>3$ and $x\ge \exp (8\cdot \sqrt{q}\cdot \ln ^3q)$. Then,

$$\begin{aligned} \theta (x,q) \ge \frac{x}{\varphi (q)} - \frac{1}{160}\cdot \frac{x}{\ln x} \end{aligned}$$

where $\varphi$ is Euler’s totient function.

Now we have everything ready to prove Lemma A.3.

### Proof of Lemma A.3

We first prove the bound for *m*. By the definition of *m* as $m={\textsf {prime\_bound}}_q(B)$, we get $\prod _{i=1}^{m-1} {\textsf {prime}}_q(i) <B$. As $\ln ({\textsf {prime}}_q(i))>1$ for every *i*, we have

$$\begin{aligned} m-1< \sum _{i=1}^{m-1} \ln ({\textsf {prime}}_q(i)) = \ln \left( \prod _{i=1}^{m-1} {\textsf {prime}}_q(i) \right) <\ln (B) \end{aligned}$$

which implies $$m<\ln (B)+1$$.

Now we prove the bound for ${\textsf {prime}}_q(m)$. For this we set

$$\begin{aligned} x= \max \left\{ \exp \left( 8\cdot \sqrt{q} \cdot \ln ^3(q) \right) ,~ \exp (q), ~2q\cdot \ln (B) \right\} . \end{aligned}$$

By Lemma A.4, we get

$$\begin{aligned} \theta (x,q)&\ge \frac{x}{\varphi (q)} - \frac{1}{160} \cdot \frac{x}{\ln x }\\&= x \left( \frac{1}{\varphi (q)} - \frac{1}{160 \cdot \ln x}\right) \end{aligned}$$

and, using $\varphi (q)\le q$ and $\ln (x)\ge q$, we have

A.1 $$\begin{aligned} \theta (x,q)&\ge x \cdot \left( \frac{1}{q } - \frac{1}{160 \cdot q} \right) \nonumber \\&\ge x \cdot \frac{1}{2\cdot q } \nonumber \\&\ge \ln B. \end{aligned}$$

Let $\ell =\max \{j ~|~{\textsf {prime}}_q(j)\le x \}$ be the index of the largest *q*-prime which is not greater than *x*. Then,

$$\begin{aligned} \prod _{i=1}^{\ell } {\textsf {prime}}_q(i)&= \exp \left( \sum _{i=1}^{\ell } \ln ({\textsf {prime}}_q(i))\right) \\&= \exp \left( \sum _{i=1}^{\infty } \llbracket {{\textsf {prime}}_q(i)\le x }\rrbracket \cdot \ln ({\textsf {prime}}_q(i))\right) \\&= \exp \left( \theta (x,q)\right) \ge B, \end{aligned}$$

where the inequality follows from (A.1). By the definition of ${\textsf {prime\_bound}}_q$, we get $m={\textsf {prime\_bound}}_q(B)\le \ell$. Hence, ${\textsf {prime}}_q(m )\le {\textsf {prime}}_{q}(\ell ) \le x$ which finishes the proof. $\square$

In the remainder we give the $\widetilde{{\mathcal {O}}}\left( (\prod _{i=1}^k \textbf{r}_i) \cdot \textrm{polylog}(M)\right)$ algorithm for the *K*-Cyclic Convolution Problem.

### Proof of Theorem 2.6

Fix a finite set $K=\{c_1,\dots , c_{\ell }\}\subseteq {\mathbb {N}}$ which is considered as a constant throughout this proof. Let integers $k, M \in {\mathbb {N}}$, integer vector $\textbf{r}\in K^k$ and functions $g,h:Z \rightarrow {\{-M,\ldots , M\}}$ where $Z={\mathbb {Z}}_{\textbf{r}_1}\times \dots \times {\mathbb {Z}}_{\textbf{r}_k}$ be an input for the *K*-Cyclic Convolution Problem.

For every $t\in [\ell ]$, let $D_t$ be the prime factors of $c_t$. We define $R= \prod _{j=1}^{k} \textbf{r}_j$ and observe that for any $\textbf{v}\in Z$ it holds that $\vert { (g \mathbin {\odot }h) (\textbf{v})} \vert \le R\cdot M^2$. Further define $B {:}{=}3 \cdot R \cdot M^2$ and $q= \prod _{c\in K} c = \prod _{t=1}^{\ell } c_t$. Assume without loss of generality that $q\ge 3$ and note that *q* depends only on the fixed finite set *K* and therefore, can be viewed as a constant.

With this notation we can formally state the algorithm.

1. Iterate over the numbers of the form $q\cdot a+1$ for $a\in \{1,2,\ldots \}$ and test for each one if it is prime. The process continues until the product of the *q*-primes exceeds *B*. Denote these numbers by $p_1,\dots ,p_m$.
2. For every $i\in [m]$ and $t\in [\ell ]$, iterate over all elements $x \in {{\mathbb {F}}}_{p_i}$ and test whether $x^{c_t}\equiv 1 \mod p_i$ and $x^{{c_t}/{d}} \not \equiv 1 \mod p_i$ for every $d\in D_t$. If so, then set *x* as the $c_t$-th root of unity in ${{\mathbb {F}}}_{p_i}$.
3. For all $i\in [m]$, use Theorem A.1 with the prime $p_i$ and appropriate roots of unity to compute the function $f^{(i)}:Z\rightarrow {\mathbb {Z}}_{p_i}$ defined by
  $$\begin{aligned} f^{(i)}(\textbf{v}){:}{=}(g \odot h)(\textbf{v}) \mod p_i~~~~~~\forall \textbf{v}\in Z. \end{aligned}$$
4. Define $P=\prod _{i=1}^{m} p_i$, we define a function $f_P:Z\rightarrow {\mathbb {Z}}_{P}$ as follows. For each $\textbf{v}\in Z$, use the Chinese Remainder Theorem (cf. Theorem A.2) to compute the value $0\le f_P(\textbf{v})<P$ such that $f_P(\textbf{v}) \equiv f^{(i)}(\textbf{v}) \mod p_i$ for all $i\in [m]$.
5. Finally, compute the function $f:Z\rightarrow {\mathbb {Z}}$ using the formula
  $$\begin{aligned} f(\textbf{v})= {\left\{ \begin{array}{ll} f_P(\textbf{v}) &{}\text { if }f_P(\textbf{v}) <\frac{P}{2} \\ f_P(\textbf{v}) - P &{} \text { if } f_P(\textbf{v}) \ge \frac{P}{2} \end{array}\right. } \end{aligned}$$
  for all $\textbf{v}\in Z$ and return *f*.

Before we move to proving the correctness, we first argue that the algorithm is well-defined. From the definition, the first step computes the first $m={\textsf {prime\_bound}}_q(B)$ *q*-primes such that $p_1 = {\textsf {prime}}_q(1), \ldots , p_m={\textsf {prime}}_q(m)$. It remains to show that, for every $i \in [m]$ and $t \in [\ell ]$, the $c_t$-th primitive root of unity in ${{\mathbb {F}}}_{p_i}$ exists. Indeed, since $c_t$ divides $p_i - 1$ (which is in turn true as $p_i \equiv 1 \mod q$ and $c_t$ divides *q*), such a root of unity exists. Moreover, as $D_t$ contains all prime factors of $c_t$, one can easily show that it actually suffices to consider only values of the form $x^{c_t/d}$ for every $d\in D_t$ to correctly decide if *x* is a primitive $c_t$-th root of unity in ${{\mathbb {F}}}_{p_i}$. The application of Theorem A.1 in the second step is possible as $\textbf{r}_j \in K=\{c_1,\ldots , c_{\ell }\}$ for every $j\in [n]$ and the roots of unity are computed by the second step.

Now we argue about the correctness of the algorithm.

### Claim A.5

For all $\textbf{v}\in Z$, we have $f(\textbf{v}) = (g \mathbin {\odot }h)(\textbf{v})$.

### Proof

As the algorithm is well defined, the third step computes, the convolution of *g* and *h* modulo $p_i$ for every $i \in [m]$.

Now fix some $\textbf{v}\in Z$. We define $b(\textbf{v}) = (h\mathbin {\odot }g)(\textbf{v}) \mod P$ and observe $0\le b(\textbf{v}) < P$. Moreover, for every $i\in [m]$ it holds that

$$\begin{aligned} b(\textbf{v}) \mod p_i = \left( (h\mathbin {\odot }g)(\textbf{v}) \mod P\right) \mod p_i = (h\mathbin {\odot }g)(\textbf{v}) \mod p_i = f^{(i)}(\textbf{v}). \end{aligned}$$

Since Theorem A.2 also guarantees the resulting number to be unique, it follows that $f_P(\textbf{v}) = b(\textbf{v})$ which implies $f_P(\textbf{v}) = (g \mathbin {\odot }h )(\textbf{v}) \mod P$.

Now we focus on the last step. By the definition of $m = {\textsf {prime\_bound}}_q(B)$, it holds that $P= \prod _{i=1}^{m}p_i \ge B = 3 \cdot R\cdot M^2$. Consider the following cases.

- In case $(g\mathbin {\odot }h) (\textbf{v}) \ge 0$ we have
  $$\begin{aligned}(g\mathbin {\odot }h) (\textbf{v})\le R\cdot M^2 < \frac{B}{2}\le P.\end{aligned}$$
  This implies that $f_P(\textbf{v}) = (g\mathbin {\odot }h)(\textbf{v}) \mod P = (g\mathbin {\odot }h)(\textbf{v}) <\frac{B}{2}$. Thus, $f(\textbf{v}) = f_P(\textbf{v}) = (g\mathbin {\odot }h)(\textbf{v})$.
- In case $(g\mathbin {\odot }h) (\textbf{v}) < 0$ it holds that
  $$\begin{aligned}(g\mathbin {\odot }h) (\textbf{v})\ge -R\cdot M^2 >-P.\end{aligned}$$
  This now implies that
  $$\begin{aligned}f_P(\textbf{v}) = (g\mathbin {\odot }h)(\textbf{v}) + P\ge P- R\cdot M^2 > \frac{P}{2}.\end{aligned}$$
  Hence, $$f(\textbf{v}) = f_P(\textbf{v}) - P = (g\mathbin {\odot }h)(\textbf{v}) + P- P = (g\mathbin {\odot }h)(\textbf{v})$$.

Hence, $f(\textbf{v}) =(g \mathbin {\odot }h) (\textbf{v})$ for all $\textbf{v}\in Z$, which concludes the proof. $\square$

From Claim A.5 we know that the algorithm is correct and the function *f* returned by the algorithm is indeed $(g\mathbin {\odot }h)$. It only remains to analyze the running time of the procedure.

### Claim A.6

The procedure terminates in time $\widetilde{{\mathcal {O}}}\left( R \cdot \textrm{polylog}(M)\right)$.

### Proof

We consider each step on its own.

1. Since prime testing can be done in polynomial time (in the representation size of the number), we can find the sequence $p_1,\dots ,p_m$ in time ${\mathcal {O}}(p_m\cdot {{\,\mathrm{\textrm{polylog}}\,}}p_m)$. By Lemma A.4, and since *q* is a constant, it follows that
  $$\begin{aligned}{} & {} p_m \le \max \left\{ \exp \left( 8\cdot \sqrt{q} \cdot \ln ^3(q) \right) ,\, \exp (q),\, 2q\cdot \ln (B) \right\} \\{} & {} = {\mathcal {O}}(\ln (B)) = {\mathcal {O}}(\log (R\cdot M)) \end{aligned}$$
  and $m\le \ln (B)+1 = \ln (3RM^2)+1$. Hence, the running time of this step is ${\mathcal {O}}(p_m\cdot {{\,\mathrm{\textrm{polylog}}\,}}p_m)= {\mathcal {O}}({{\,\mathrm{\textrm{polylog}}\,}}(R\cdot M))$.
2. For each $i\in [m]$ and $t\in [\ell ]$, in Step 2. of the algorithm we iterate over $p_i$ values and check $\vert {D_t} \vert$ values. Since $D_t$ are the prime factors of $c_t$ (and hence $|D_t|$ is a constant), this takes time ${\mathcal {O}}(p_i {{\,\mathrm{\textrm{polylog}}\,}}p_i)$ which can be bounded by ${\mathcal {O}}({{\,\mathrm{\textrm{polylog}}\,}}(R\cdot M))$. Since $m\le \log (3\cdot R\cdot M^2)+1$ and $\ell$ is a constant, the overall running time of the step is ${\mathcal {O}}({{\,\mathrm{\textrm{polylog}}\,}}(R\cdot M))$.
3. By Theorem A.1, the number of arithmetic operations required to compute $f^{(i)}$ is ${\mathcal {O}}(R\cdot \log (R))$. Since each arithmetic operation is performed in ${{\mathbb {F}}}_{p_i}$, the total time spent to compute $f^{(i)}$ is
  $$\begin{aligned}{} & {} {\mathcal {O}}(R\cdot \log (R)\cdot \log ^2(p_i)) = {\mathcal {O}}(R\cdot \log (R)\cdot \log ^2( \log (R \cdot M ) ))\\{} & {} = {\mathcal {O}}(R\cdot {{\,\mathrm{\textrm{polylog}}\,}}(R\cdot M)), \end{aligned}$$
  where the first equality holds because $p_i\le p_m={\mathcal {O}}(\log (R\cdot M))$. Finally, as $m={\mathcal {O}}(\log (R\cdot M))$, the overall computation time of this step is $m\cdot {\mathcal {O}}(R\cdot {{\,\mathrm{\textrm{polylog}}\,}}(R\cdot M) ={\mathcal {O}}(R\cdot {{\,\mathrm{\textrm{polylog}}\,}}(R\cdot M))$.
4. As we iterate over all *R* values from *Z* and by Theorem A.2, this computation can be done in time ${\mathcal {O}}(R\cdot (\log P)^2)$. Since $\log P \le m \cdot p_m \le {\mathcal {O}}({{\,\mathrm{\textrm{polylog}}\,}}(R\cdot M))$ the overall running time of this step is ${\mathcal {O}}(R\cdot {{\,\mathrm{\textrm{polylog}}\,}}(R\cdot M))$.
5. As we again iterate over all elements from *Z*, the computation time of this step is ${\mathcal {O}}(R\cdot {{\,\mathrm{\textrm{polylog}}\,}}P)= {\mathcal {O}}(R\cdot {{\,\mathrm{\textrm{polylog}}\,}}(R\cdot M))$ where we use $P={\mathcal {O}}({{\,\mathrm{\textrm{polylog}}\,}}(R\cdot M))$.

As the running time of each step is at most $\widetilde{{\mathcal {O}}}\left( R \cdot \textrm{polylog}(M)\right)$, the overall running time of the algorithm is $\widetilde{{\mathcal {O}}}\left( R \cdot \textrm{polylog}(M)\right)$. $\square$

The proof now follows by Claim A.5 and A.6. $\square$

## Footnotes

1 We use $\widetilde{{\mathcal {O}}}(x)=x\cdot {{\,\mathrm{\textrm{polylog}}\,}}(x)$ notation to hide polylogarithmic factors. We assume that *M* is the maximum absolute value of the integers on the input.

2 We provide a formal definition of $\oplus _f$ in Sect. 2.

3 This observation was brought to our attention by Nederlof [27].

4 It is a special case with $D= \{0,1\}$, $\textbf{v}= 0^n$ and $f(x,y) = x \cdot y$

5 We use $\varepsilon$ to denote the vector of length 0.

6 Note that there might be multiple $r \in R$ with $\lambda _f(r)=(t_j,t_{j+1})$.
